# ELE: Estimating tissue‐specific long noncoding RNA gene essentiality using graph neural networks

**DOI:** 10.1002/qub2.70053

**Published:** 2026-09-21

**Authors:** Wan‐Ting Shi, Ying‐Dong Liu, Xiu‐Jun Gong, Pu‐Feng Du

**Affiliations:** ^1^ School of Computer Science and Technology Tianjin University Tianjin China; ^2^ College of Intelligence and Computing Tianjin University Tianjin China; ^3^ School of Artificial Intelligence Tianjin University Tianjin China; ^4^ Key Laboratory of Systems Bioengineering (Ministry of Education) Tianjin University Tianjin China

**Keywords:** essential gene, graph neural network, lncRNA‐protein‐protein interaction network

## Abstract

A gene is essential if its loss of function results in lethality, reduced fitness, or disease. Essential genes have attracted increasing attention due to their vital functions in biological systems. Many efforts have been made to find essential protein‐coding genes. In complex organisms, the majority of the genome does not encode proteins. Noncoding genes play vital roles in many biological processes. Investigating the essentiality of noncoding genes is necessary. We present ELE (estimating long noncoding RNA essentiality), the first computational model for finding essential long noncoding RNA (lncRNA) genes in a tissue‐specific context. ELE integrates lncRNA‐protein–protein interaction (LPPI) network structure with genomic and epigenomic features to predict essential lncRNA genes. With ELE, we identified tissue‐specific essential lncRNA genes in human and mouse. Tissue‐specific essential lncRNA genes are more involved in physiological context‐dependent cellular functions. Moreover, we found that essential lncRNA genes tend to interact more with essential proteins and essential microRNAs (miRNAs). ELE serves as an effective framework for deciphering lncRNA gene essentialities, offering a set of tissue‐dependent candidates for further validation. We have deposited all datasets and codes for reproducing ELE results in a GitHub repository (swt1024/ELE).

## 1 INTRODUCTION

A gene is essential when the loss of its function compromises the viability of an individual or results in a profound loss of fitness [1]. This concept applies to both protein‐coding genes and noncoding RNA genes. Elucidating gene essentiality not only facilitates the understanding of pathogenic mechanisms in genetic disorders [2] but also helps the development of therapeutic chemicals [3].

Genome‐wide screening methods, such as CRISPR/Cas9 and RNA interference (RNAi), have been widely applied to identify essential genes [4, 5, 6]. However, these experimental approaches are costly and labor‐intensive. Therefore, computational predictions have emerged as effective and efficient alternative approaches.

Many efforts have been made to identify essential protein‐coding genes computationally [7, 8, 9, 10]. Databases for essential genes, such as DEG [11] and OGEE [12], also focus mainly on protein‐coding genes. However, the ENCODE project [13] revealed that, although only 1%–2% of the human genome encodes proteins, up to 80% of human genome is biochemically active. Noncoding RNAs, particularly long noncoding RNAs (lncRNAs), play significant roles in gene expression regulation [14, 15], cell differentiation, and individual development [15, 16]. Despite their importance, the exploration of lncRNA gene essentiality remains preliminary, compared to that of protein‐coding genes. Early attempts, such as the gene importance calculator (GIC) [17] method and the XGEP study [18], largely rely on transferring sequence and expression profile‐based models, which are originally trained on protein‐coding genes, to lncRNA genes.

LncRNA‐protein interaction (LPI) is a key mechanism, through which lncRNA genes exert their functions. Therefore, lncRNA‐protein interactions can provide important information in finding essential lncRNA genes. Xin et al. proposed the systematic gene importance index (SGII) method [19], which prioritizes lncRNA genes based on their centrality in the lncRNA‐protein–protein interaction (LPPI) network. This work indicates that the essentiality of a lncRNA gene is correlated with structural features of the LPPI network [19]. However, the SGII scoring scheme is too simple to capture complex network structural information. Zhang et al. developed iEssLnc [20], a random walk‐based method that extracts complex structural features from the LPI network. The iEssLnc method further supports the idea that the essentiality of a lncRNA gene is related to its local structural pattern in the LPI network.

Although these methods successfully utilized network structural features, they failed to incorporate the intrinsic genomic and epigenomic features of lncRNA genes [2]. These features are proved to be useful in finding essential coding genes [7]. In addition, substantial evidence indicates that gene essentiality is a conditional trait, depending on biological backgrounds and environmental contexts [21]. However, for lncRNA genes, current computational methods quantify their essentialities without considering any biological or environmental context. To bridge this gap, we propose ELE (estimating long noncoding RNA essentiality), a graph neural network‐based method for predicting tissue‐specific essential lncRNA genes. With ELE, we identified a set of tissue‐specific essential lncRNA genes in human and mouse. These essential lncRNA genes present special functional patterns that are related to their cellular environments. Our results demonstrated that ELE can construct a high‐resolution genome‐wide map of lncRNA gene essentialities across various tissues in both human and mouse.

## 2 RESULTS

### 2.1 Overview of the ELE framework

The workflow of ELE is illustrated in Figure 1. The ELE framework is composed of three modules, including a network construction module, a feature generation module and a predictor module. The network construction module combines lncRNA–protein interactions with protein–protein interactions to construct an LPPI network. The feature generation module utilizes various types of features of lncRNA genes and proteins, including genomic sequences, gene expression profiles, functional annotations, evolutionary scores, and epigenomic modification tracks. The HinSAGE [22] method was used to integrate all these features with the LPPI network local structural feature to produce an embedding for every lncRNA gene in the LPPI network. The predictor module applied supervised machine learning algorithms, such as the support vector machine (SVM) or the multilayer perceptron (MLP), to predict essentiality for all lncRNA genes. For model training, positive samples were obtained from the essential lncRNA annotations in the dbEssLnc 2.0 database [23], whereas negative samples were preferentially selected based on GIC scores. Because the epigenomic modifications and gene expressions are tissue‐specific, ELE identifies essential lncRNA genes in a tissue‐specific way. We used ELE to analyze three human tissues (left ventricle, left lung, and stomach) and 3 mouse tissues (heart, lung, and forebrain).

**FIGURE 1:**
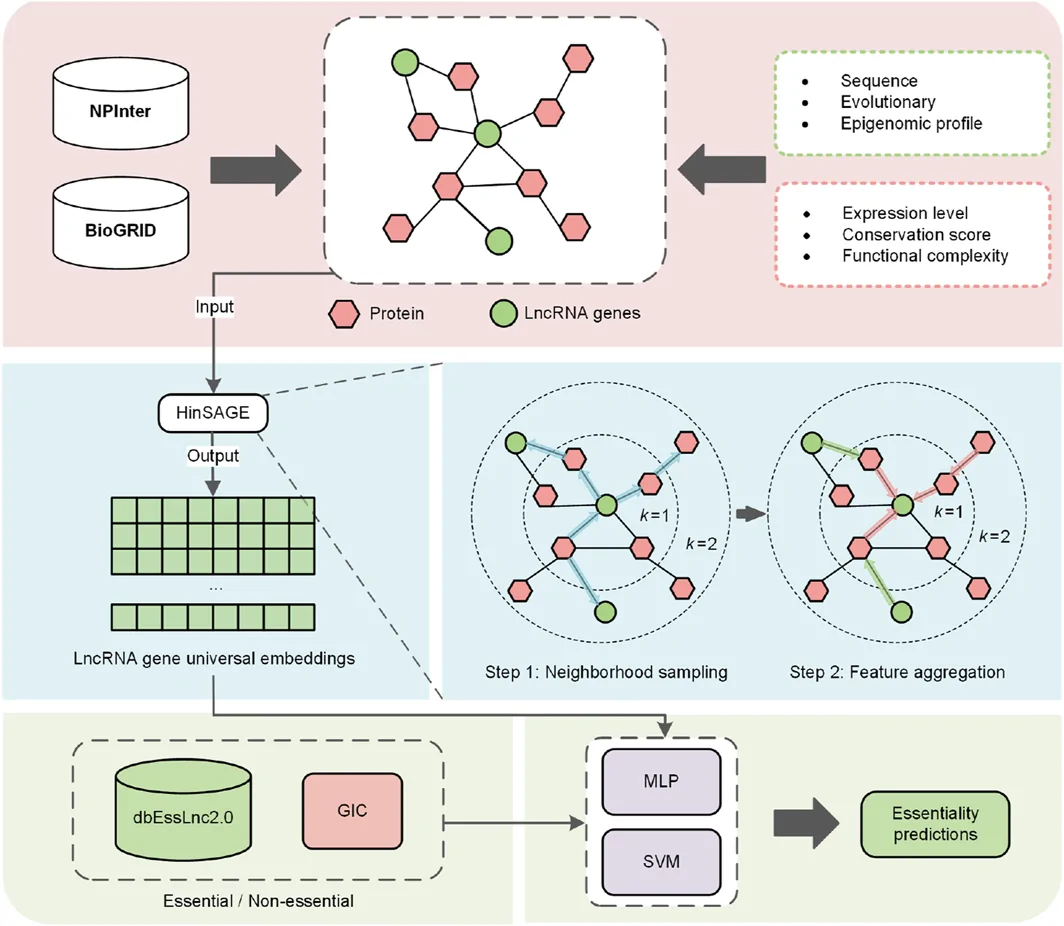
Workflow of the ELE. After constructing the LPPI networks, the raw genomic and epigenomic features were collected. These raw features were aggregated by HinSAGE, producing universal representations for lncRNA genes. Essentiality marks were annotated according to dbEssLnc2.0 records. SVM and MLP models were trained on these universal representations. ELE, estimating long non‐coding RNA essentiality; LPPI, lncRNA‐protein‐protein interaction; MLP, multilayer perceptron; SVM, support vector machine.

### 2.2 Predictive performance analysis

SVM and MLP models were trained to find essential lncRNA genes. Due to the different dataset sizes between the human and the mouse, we applied the 10‐fold cross‐validation on all human tissues and the leave‐one‐out cross validations on all mouse tissues. We used the area under receiver operating characteristic curve (AUROC) and the area under precision–recall curve (AUPR) to measure the predictive performance of ELE (Figure 2). SVM models performed better than MLP models on human tissues, whereas MLP models demonstrated superior performances on mouse tissues.

**FIGURE 2:**
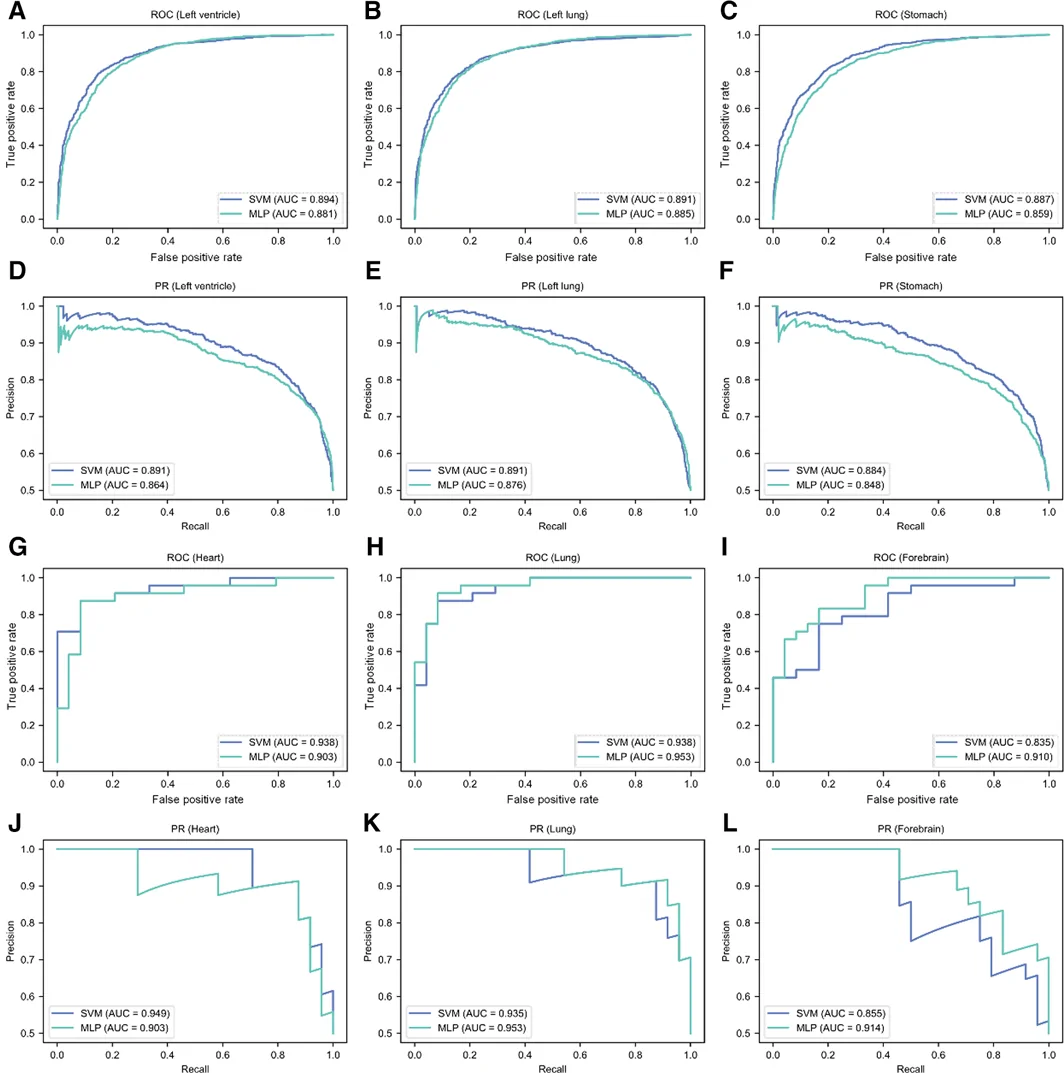
Predictive performance analysis of ELE across different tissues. (A–C) ROC (receiver operating characteristic) curves for human tissues; (D–F) PR (precision‐recall) curves for human tissues; (G–I) ROC curves for mouse tissues; (J–L) PR curves for mouse tissues. AUC, area under curve; ELE, estimating long non‐coding RNA essentiality; MLP, multilayer perceptron; SVM, support vector machine.

We calculated additional performance measures (Supporting Information S1: Table S1), including sensitivity (Sen), specificity (Spe), positive predictive value (PPV), F_1_‐score (F_1_), accuracy (Acc), and Matthews correlation coefficient (MCC). We compared ELE against other state‐of‐the‐art methods (iEssLnc, SGII and GIC) on our benchmarking datasets (human left ventricle and mouse heart) using additional performance measures. ELE demonstrated superior performance over all existing methods (Figure 3).

**FIGURE 3:**
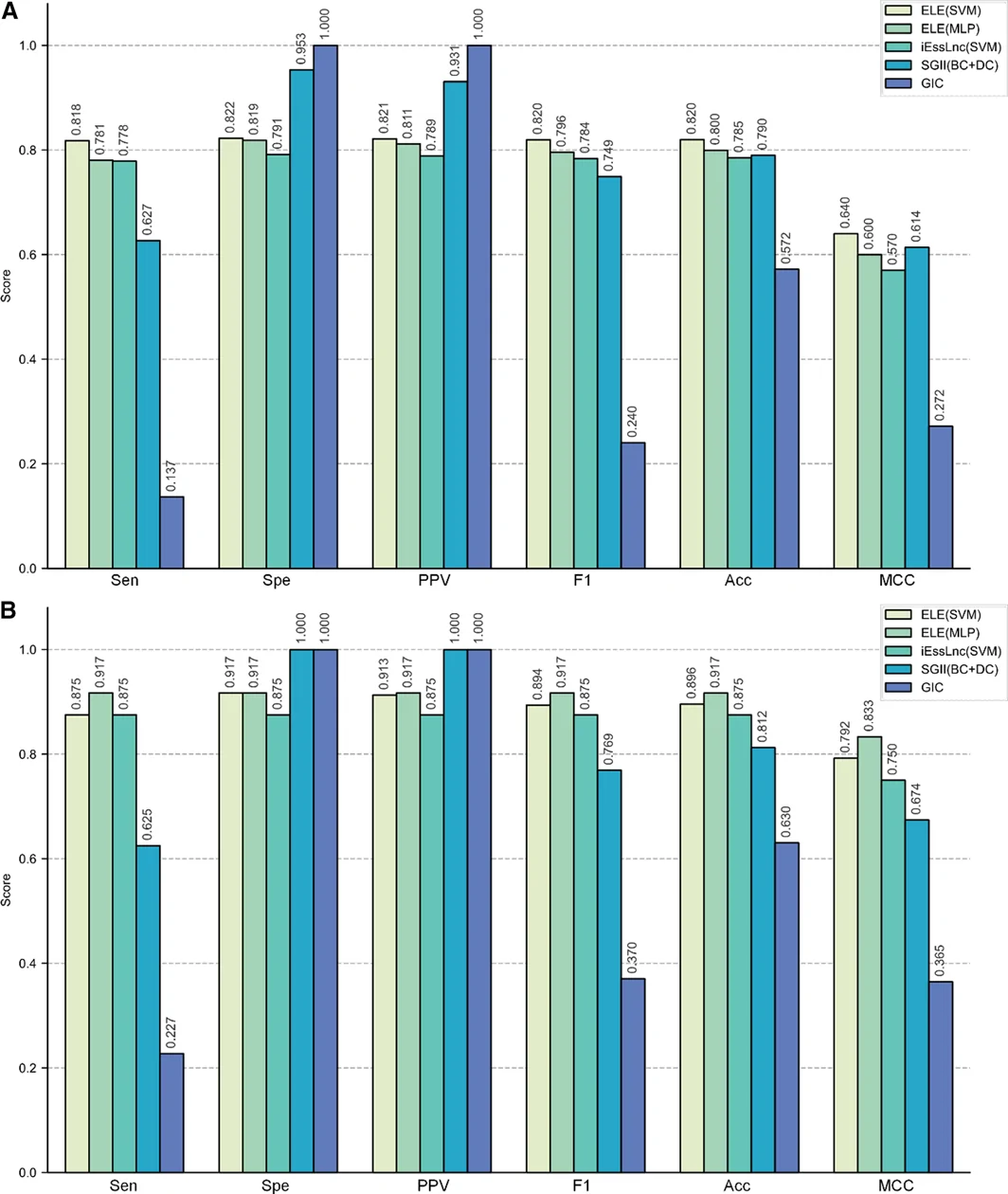
Performance comparisons between ELE and other state‐of‐the‐art methods (iEssLnc, SGII, and GIC). (A) Performance comparison in human left ventricle. (B) Performance comparison in mouse heart. Acc, accuracy; BC, betweenness centrality; DC, degree centrality; ELE, estimating long non‐coding RNA essentiality; F_1_, F_1_‐score; MCC, Matthews correlation coefficient; MLP, multilayer perceptron; PPV, positive predictive value; Sen, sensitivity; Spe, specificity; SVM, support vector machine.

To ensure that our predictions reflect genuine associations between lncRNA essentiality and the biological features, we performed a randomization test. We randomly permuted the essentiality labels 1000 times for each tissue. Following the original protocol, we performed cross‐validation on each randomized dataset. For every tissue, the AUROC value on the original data consistently outperformed all 1000 randomizations. These results indicate that the performance of our model is not produced by random chances (*p* < 0.001, randomization test), but reflects real associations between lncRNA essentiality and underlying features.

### 2.3 Essential lncRNA gene enrichment analysis

Due to the limited availability of high‐quality data on nonessential lncRNA genes, reliable negative labels are difficult to obtain directly. Consequently, we utilized the GIC score, a validated metric for lncRNA essentiality, to preferentially select lncRNA genes with lower scores as negative samples. However, given the potential uncertainty associated with these negative samples, traditional performance metrics (such as Acc and AUROC) may not fully reflect the model’s practical performance.

To evaluate ELE’s ability to prioritize known essential lncRNA genes, we applied gene set enrichment analysis (GSEA) [24] on the ranked predictions generated by ELE. By calculating enrichment scores, we evaluated the overrepresentation of known essential lncRNA genes within high‐scoring regions. We further compared ELE with iEssLnc and GIC. As shown in Figure 4, the ELE model with SVM achieved the highest enrichment score (*p* < 0.001, Kolmogorov‐Smirnov test), outperforming both iEssLnc and GIC.

**FIGURE 4:**
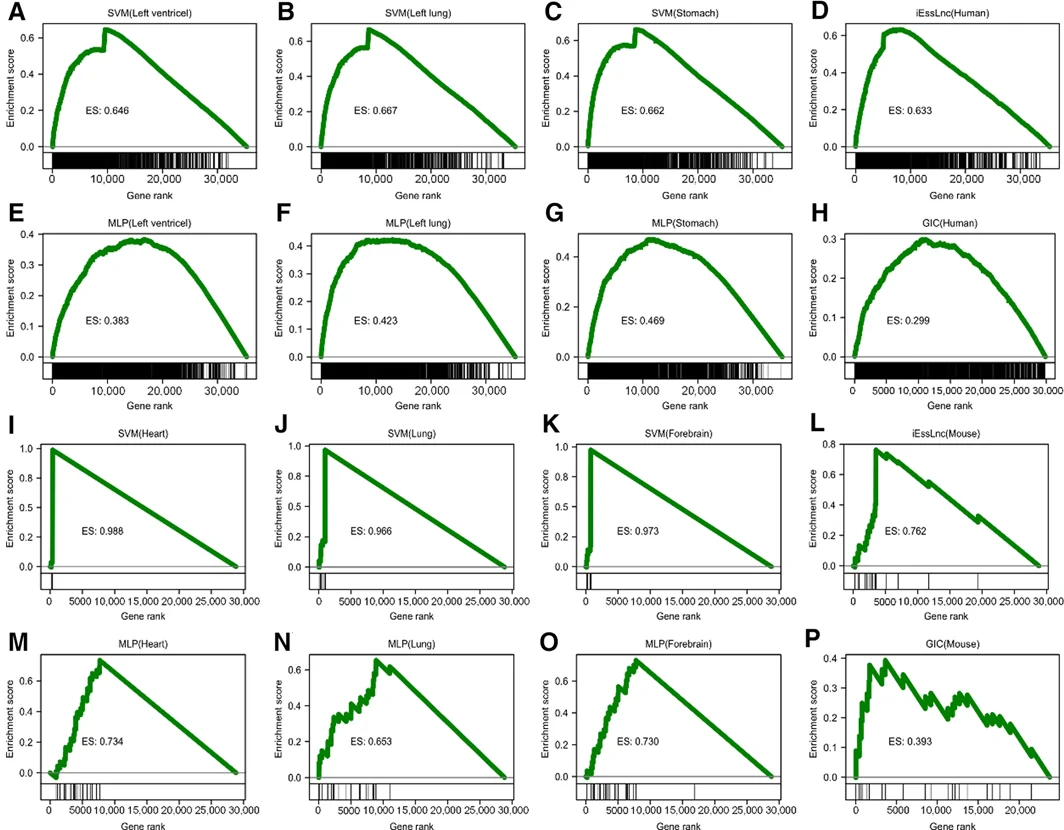
Enrichment analysis of ELE models, with comparisons to other state‐of‐the‐art methods (iEssLnc and GIC). For each panel, the curve represents the running enrichment score across the ranked gene list. The short vertical bars in the pseudo‐gel indicate the positions of essential lncRNA genes in the ranked list. The peak of the curve corresponds to the final enrichment score. (A–C) and (E–G) Enrichment analysis of ELE with SVM and MLP, respectively, on the human dataset. (D) Enrichment analysis of the iEssLnc method on the human dataset. (H) Enrichment analysis of GIC method on the human dataset. (I–K) and (M–O) Enrichment analysis of ELE with SVM and MLP, respectively, on the mouse dataset. (L) Enrichment analysis of iEssLnc method on the mouse dataset. (P) Enrichment analysis of the GIC method on the mouse dataset. ELE, estimating long non‐coding RNA essentiality; ES, enrichment scores; MLP, multilayer perceptron; SVM, support vector machine.

### 2.4 Tissue‐specific essential lncRNA gene identification

We used ELE to annotate the essentiality of all lncRNA genes within our LPPI networks. For each tissue, we retained only the prediction results shared by both SVM and MLP predictors. For human tissues, ELE identified 16,817 essential lncRNA genes in the left ventricle, 16,177 in the left lung, and 15,158 in the stomach. For mouse tissues, ELE predicted 6883 essential lncRNA genes in the heart, 10,068 in the lung, and 7039 in the forebrain. These figures significantly exceeded our expectations.

According to Ref. [25], essential protein‐coding genes typically account for 10%–40% of all protein‐coding genes. Assuming a similar proportion for lncRNA genes, nonessential lncRNA genes should be much more than essential ones in a genome‐wide screening. Because ELE was trained on a balanced dataset, above numbers are more like a result of an overwhelming number of false positives.

To control the number of false positives in the genome‐wide screening context, we examined the betweenness centrality (BC) score [26] distributions of all lncRNA genes versus the essential lncRNA genes, which are identified by ELE (Figure 5 and Supporting Information S1: Figure S1). Intuitively, the essential lncRNA genes had a very similar BC score distribution to that of all lncRNA genes. This was observed across all tissues in both the human and the mouse. However, according to Ref. [19], BC score distribution of essential lncRNA genes should be different from that of all lncRNA genes.

**FIGURE 5:**
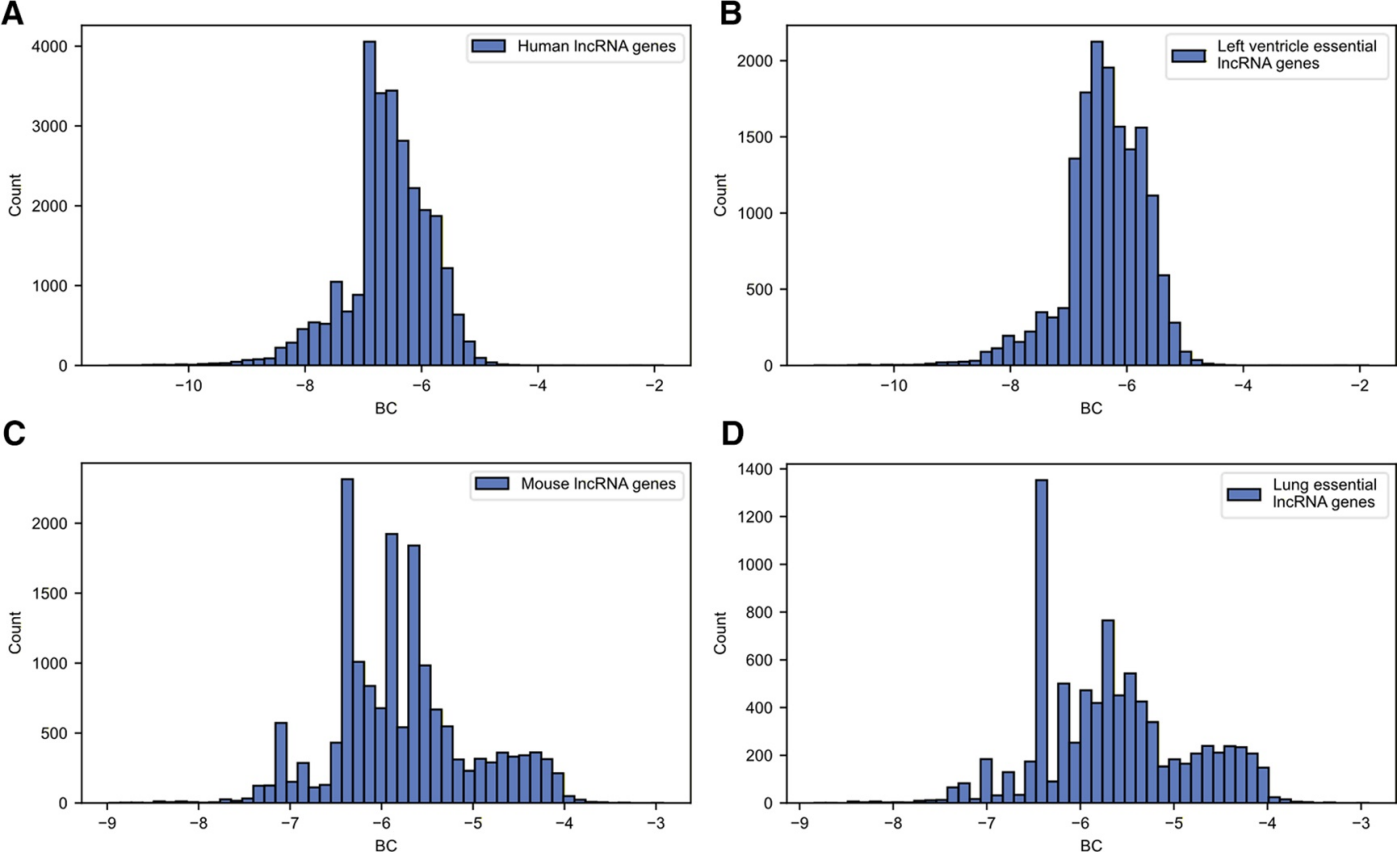
BC distributions of human and mouse lncRNA genes across the LPPI network. (A, B) Distributions in the human dataset, showing (A) all lncRNA genes and (B) predicted essential lncRNA genes in the left ventricle. (C, D) Distributions in the mouse dataset, showing (C) all lncRNA genes and (D) predicted essential lncRNA genes in the lung. BC, betweenness centrality.

Based on this observation, we filtered ELE predictions using BC scores. All essential lncRNA genes, which are predicted by ELE, were ranked in descending order according to their BC scores. We retained the top 40% of each human tissue and the top 60% of each mouse tissue. When tied BC scores were found at the threshold, all tied lncRNA genes were retained (Supporting Information S1: Table S2). We finally identified 6727 (left ventricle), 6471 (left lung), and 6063 (stomach) essential lncRNAs in the human, and 4154 (heart), 6202 (lung), and 4243 (forebrain) in the mouse. Figure 6 presents the number of essential lncRNA genes that are shared by different tissues. We found 4796 essential lncRNA genes that are shared by all three human tissues, and 1554 by all three mouse tissues. We termed these genes the “common essential lncRNA genes.” The predicted tissue‐specific essential lncRNA genes, nonessential lncRNA genes, and common essential lncRNA genes have been deposited in the Zenodo repository.

**FIGURE 6:**
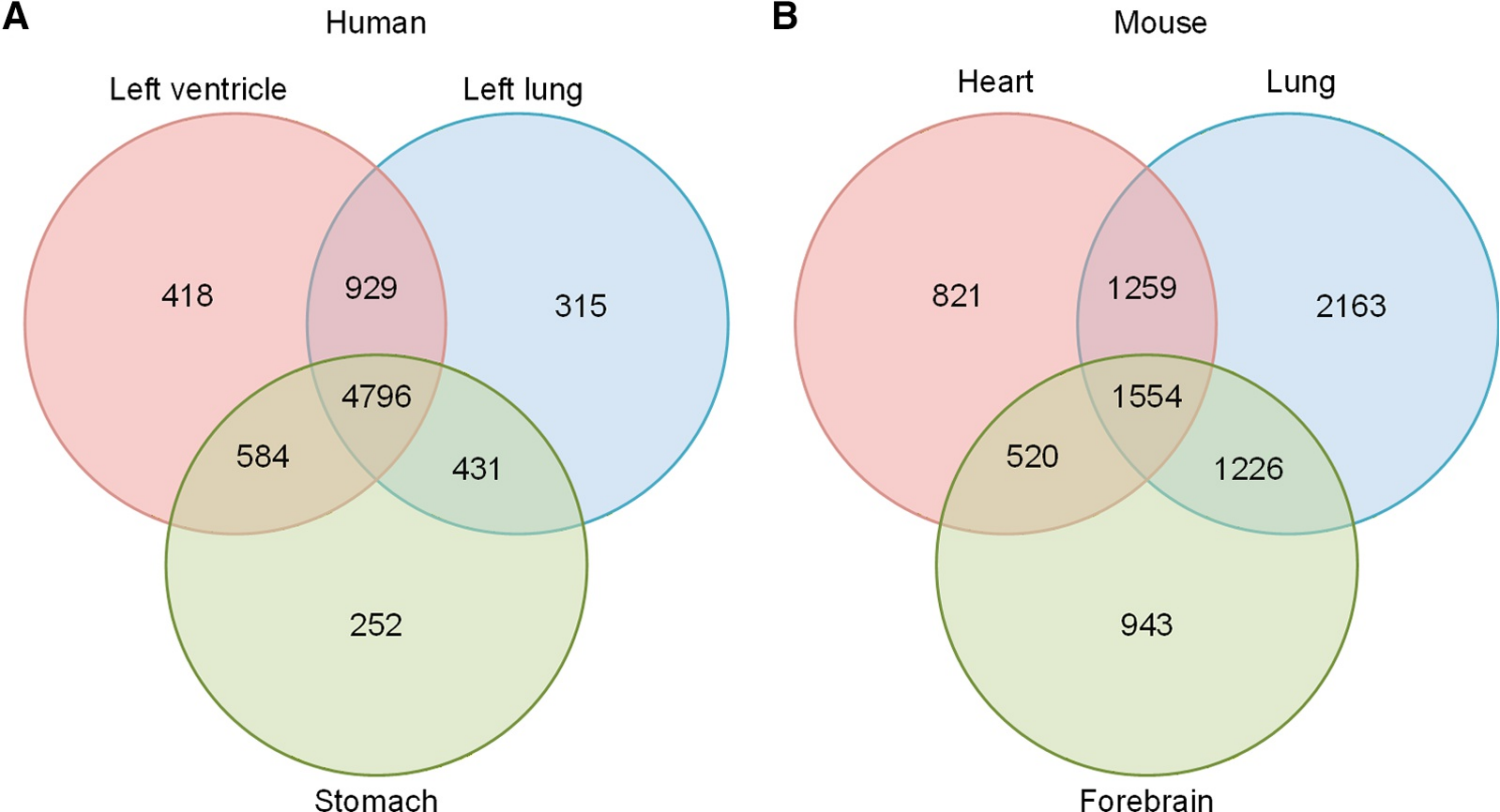
Tissue‐specific and common essential lncRNA genes. (A) Venn diagram showing the overlap of essential lncRNA genes identified in human left ventricle, left lung, and stomach. (B) Overlap of essential lncRNA genes identified in mouse heart, lung, and forebrain.

### 2.5 Functional analysis of tissue‐specific essential lncRNA genes

Considering that gene essentiality is a context‐dependent trait, we analyzed functional patterns of tissue‐specific essential lncRNA genes. We applied the DAVID pipeline [27] to annotate functions of all essential lncRNA genes, including tissue‐specific ones and common ones. We kept gene ontology (GO) annotations with a false discovery rate < 0.05 and a fold enrichment > 1. The top five enriched terms in each ontology are listed in Supporting Information S1: Table S3. If less than five enriched terms were found, all of them were listed.

Across all datasets, “protein binding” emerged as the most enriched molecular function term. This is reasonable because the design of ELE relies on the LPPI network structural features. The common essential lncRNA genes are predominantly enriched in fundamental housekeeping functions, such as “protein binding” and “RNA processing,” regardless of whether in the human or the mouse. Tissue‐specific essential lncRNA genes are linked to the unique physiological identities of each tissue.

For example, mouse lung‐specific essential genes present prominent enrichment for “zinc ion binding” (GO:0008270, *p* < 10^−21^). This observation resonates with the critical physiological demand of pulmonary tissue. Given that the lung is constantly subjected to oxidative stress and environmental insults [28], zinc is indispensable. Accumulating evidence suggests that zinc orchestrates basal defense functions through its anti‐inflammatory, antioxidant, and antiviral properties, thereby serving as a key regulator of pulmonary homeostasis [29].

Human left lung‐specific essential lncRNA genes were significantly enriched in “rhythmic process” (GO:0048511, *p* < 10^−5^). Substantial evidence indicates that lung function is governed by circadian rhythms [30]. The disruption of circadian molecular clock is closely implicated in the pathogenesis of a spectrum of chronic respiratory disorders, including lung cancer [30].

In the human left ventricle, “ubiquitin‐dependent protein catabolic process” (GO:0006511, *p* < 10^−7^) was identified as the second most significant biological process term. This enrichment suggests the critical dependence of ventricular tissue on the ubiquitin–proteasome system for protein quality control. Because cardiomyocytes are terminally differentiated and lack the ability to dilute toxic protein aggregates through cell division [31], UPS‐mediated proteolysis constitutes a central component of the cardiac protein quality control machinery, serving as a primary defense against the accumulation of misfolded or damaged proteins [31].

### 2.6 Interactions between essential coding and essential noncoding genes

With the result of ELE predictions, it is interesting to see if essential lncRNA genes interact more with essential protein‐coding genes. We retrieved the human and the mouse essential protein‐coding gene records from the OGEE database [12]. We quantified the number of essential lncRNA genes interacting with essential protein‐coding genes and compared this number against that with the nonessential protein‐coding genes (Supporting Information S1: Table S4). We found that essential lncRNAs interact significantly more with essential proteins (*p* < 10^−95^, chi‐square test). This significance was constantly observed across all tissues in both the human and the mouse.

Figure 7 shows representative interactions with essential proteins of two essential lncRNA genes: human *MALAT1* and mouse *Xist*. *MALAT1* is a nuclear‐retained lncRNA that plays a pivotal role in alternative splicing and gene regulation [32]. It interacts with several pre‐mRNA processing factors, including PRPF8, PRPF6, and PRPF31, which are core components of the spliceosome [33, 34, 35] (Figure 7A). *Xist* is an essential regulator of X‐chromosome inactivation. It mediates targeted gene silencing through interactions with multiple essential protein factors, such as Myef2, Hnrnpm, and Celf1 [36] (Figure 7B).

**FIGURE 7:**
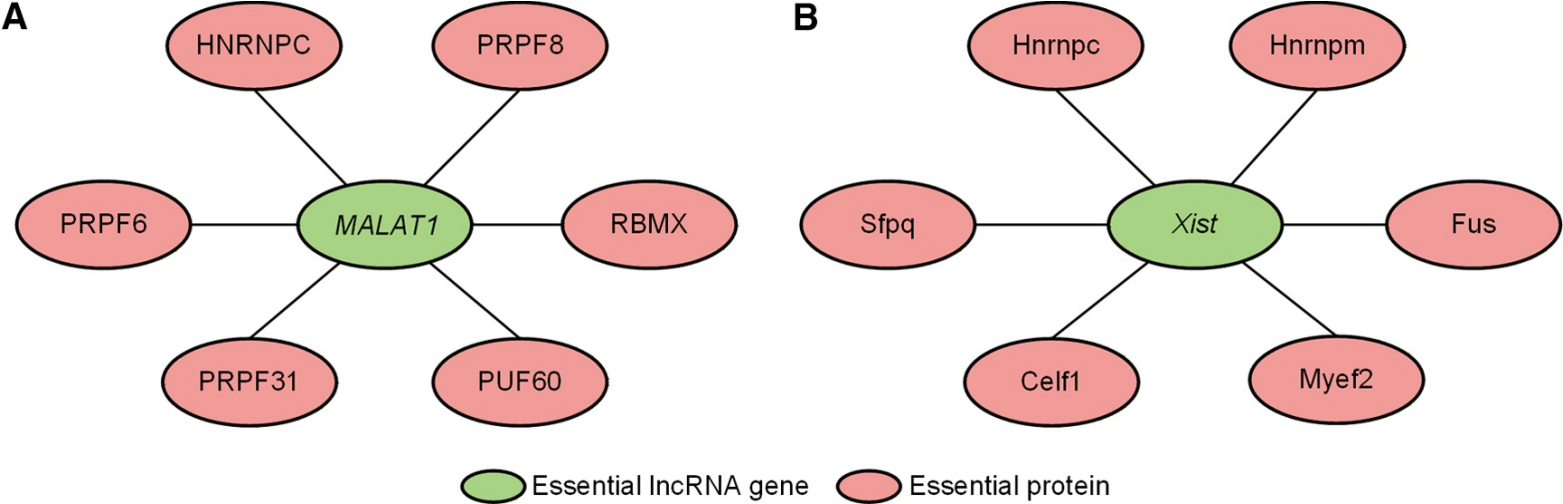
Representative interaction networks between essential lncRNA genes and essential proteins. (A) Human dataset. The network contains the human common essential lncRNA gene *MALAT1* and six interacting essential proteins. (B) Mouse dataset. The network contains the mouse common essential lncRNA *Xist* and six interacting essential proteins.

### 2.7 Interactions with essential microRNAs (miRNAs)

LncRNAs can act as molecular sponges to sequester miRNAs and indirectly regulate transcription and translation [37]. We analyzed the interactions between predicted essential lncRNA genes and known essential miRNAs. Specifically, we collected the human and the mouse lncRNA–miRNA interaction data from the NPInter database [38] and curated known essential miRNAs from existing literature, comprising 45 human [39] and 90 mouse [40, 41, 42] miRNAs. We quantified the number of interacting essential miRNAs for each essential lncRNA genes and compared with nonessential lncRNA genes. We found that essential lncRNA genes interacted with significantly more essential miRNAs than nonessential lncRNA genes (*p* < 10^−2^, Mann−Whitney *U* test). This pattern was consistently observed across all examined tissues in the human but reached significance only in the mouse in the lung.

### 2.8 Conservation analysis of tissue‐specific essential lncRNA genes

Essential genes generally exhibit higher sequence conservation due to their functional indispensability [43]. We analyzed the evolutionary conservation of the predicted essential lncRNA genes. LncRNA genes typically exhibit low overall sequence conservation, with higher constraint often restricted to specific functional elements such as promoters, exon structures, and splice sites [16]. To account for this localized conservation, we evaluated both the mean and maximum phastCons and phyloP scores of essential lncRNA genes and compared these metrics with background lncRNA genes. The results revealed that although the mean conservation scores remained comparable between the two groups, the maximum scores of essential lncRNA genes were significantly higher than those of the background genes (*p* < 10^−107^, Mann−Whitney *U* test).

Furthermore, we utilized homologous gene annotations from the Ensembl BioMart [44] to quantify the conservation of essential lncRNA genes between the human and the mouse. We identified 235 orthologous essential gene pairs between the human left ventricle and the mouse heart, 344 pairs between the human left lung and mouse lung and 92 pairs between the common essential lncRNA genes in both species.

## 3 DISCUSSION

Finding essential noncoding RNA genes is an important step toward defining the minimal genome of a complex organism. In this study, we developed ELE, a computational framework for identifying tissue‐specific essential lncRNA genes. ELE integrates various kinds of genomic and epigenomic information with structural features of the LPPI network. Compared with previous methods that mainly rely on network topology or transferred models from protein‐coding genes, ELE provided a context‐aware strategy for lncRNA essentiality estimation in both the human and the mouse, and outperformed existing methods in overall predictive ability. The randomization test further demonstrated that ELE captures nonrandom signals associated with lncRNA gene essentiality.

We acknowledge that the genome‐wide predictions of ELE likely contained a relatively high false‐positive rate. A likely explanation is that ELE was trained on a balanced dataset, whereas nonessential lncRNA genes account for a much larger proportion of lncRNA genes in the genome. The filtering step based on BC score partially alleviated this problem and improved the biological plausibility of the final predictions of ELE. Because BC scores represent more global network structural information [26], this result also suggests that the HinSAGE module in the ELE fails to capture global structural features of the LPPI network.

We further examined the predicted essential lncRNA genes in terms of functional patterns, molecular interactions, and evolutionary conservation. There was a marked difference in the functional patterns of common and tissue‐specific essential lncRNA genes. Common essential lncRNA genes were mainly enriched in housekeeping‐related functions, such as protein binding and RNA processing. Tissue‐specific essential lncRNA genes were associated with functions relevant to the physiological identity of individual tissues. In addition, essential lncRNAs tended to interact more frequently with essential proteins and more known essential miRNAs, suggesting that essential lncRNA genes were closely associated with other essential biomolecules. We also observed that essential lncRNA genes showed stronger local sequence conservation, which is consistent with the known evolutionary properties of lncRNA genes. Moreover, the presence of homologous essential lncRNA gene pairs between corresponding human and mouse tissues suggests that a subset of these genes may be evolutionarily conserved across species.

Future work of ELE may focus on improving the modeling of global network structure. It will also be reasonable to define the essential modules within the LPPI network that include both protein‐coding genes and lncRNA genes.

## 4 CONCLUSION

Overall, ELE provided a flexible framework for essential lncRNA prediction. By using ELE, we constructed the first tissue‐specific map for lncRNA gene essentialities in the human and the mouse. Functional analysis indicated that tissue‐specific essential lncRNA genes tend to exhibit functional signatures relevant to the physiological context of the tissue. In addition, the results of ELE indicated that essential lncRNAs tend to interact more with essential proteins and essential miRNAs. Our analysis suggests that ELE is a powerful tool for exploring noncoding gene essentiality, providing valuable context‐aware essential lncRNA genes for further validation.

## 5 MATERIALS AND METHODS

### 5.1 LPPI network construction

We obtained lncRNA‐protein interactions from the NPInter database v5.0 [38], protein–protein interactions from the BioGRID database v4.0 [45]. Only “binding” type lncRNA‐protein interactions from a single organism were kept. Similarly for protein‐protein interactions, only physical interactions from a single organism were kept.

All protein names were mapped to their official gene symbols to remove redundancy. We removed redundant lncRNA records in our datasets by two steps. The first step was to map all transcript identifiers to gene IDs using annotations from the NONCODE [46, 47]. We preferred the gene ID as the primary identifier. When a gene ID was not available, the gene symbol was used as a fallback. If an lncRNA cannot be identified by a gene ID nor a gene symbol, this lncRNA was removed from the dataset, along with all corresponding interactions. The second step was to merge overlapping records. We retrieved genomic coordinates for each lncRNA gene in our dataset by searching their primary identifiers sequentially against the NONCODE v6, the NONCODE v5, and the Ensembl [48] databases. All genomic coordinates were converted to be based on GRCh38 (human) assembly or GRCm38 (mouse) assembly. If two lncRNA genes had an overlap with a length exceeding 50% of the shorter one’s length, these two lncRNA genes were merged as one. The new lncRNA gene took the union of coordinate ranges of both original ones, along with all interactions. We iteratively merged all overlapping lncRNA genes in our datasets.

The lncRNA–protein interactions and the protein–protein interactions were integrated by mapping protein records that represent same molecules. The LPPI network was represented as an undirected graph, where duplicated edges and self‐loops were all removed. The statistics for the LPPI networks were recorded in Supporting Information S1: Table S5.

### 5.2 LncRNA gene essentiality annotations

We retrieved lncRNA essentiality annotations from the dbEssLnc2.0 database [23]. The dbEssLnc2.0 database is a comprehensive database of experimentally validated essential lncRNAs. The essentiality records in the dbEssLnc2.0 database were mapped on the LPPI networks by matching the primary identifiers of lncRNA genes. If an lncRNA gene in the LPPI networks could be mapped directly by a record in the dbEssLnc2.0 database, this lncRNA gene was marked as essential. For lncRNA genes, which were produced by merging more than one original lncRNA genes, they were marked as essential if at least one of the original lncRNA genes could be mapped by a record in the dbEssLnc2.0 database. A total of 1372 essential lncRNA genes were marked in the LPPI networks for human tissues, 24 for mouse tissues (Supporting Information S1: Tables S6 and S7).

### 5.3 LncRNA and protein raw features

Various types of genomic and epigenomic raw features were collected for lncRNAs and proteins in the LPPI networks (Supporting Information S1: Table S8). The raw features for lncRNAs fell into three categories: sequence statistics, evolutionary statistics, and epigenomic profile statistics. The sequence statistics category contained four features: (1) length, the total length of the lncRNA gene; (2) GC content, the proportion of guanine and cytosine bases; (3) CpG count, the total number of CG dinucleotides; and (4) CpG island count, the count of regions defined by at least two consecutive CG dinucleotides [49]. The evolutionary statistics category contained statistics from the phyloP and phastCons scores [50]. We retrieved phyloP and phastCons scores, which are derived from vertebrate whole‐genome alignments (100 vertebrates for human; 60 for mouse), from the UCSC Genome Browser [51]. The mean value and the maximal value of both scores within each lncRNA gene were used as features. The epigenomic profile statistics category reflected cell‐type‐specific features of lncRNA genes [52]. For each tissue, we retrieved ChIP‐seq profiles for DHS (DNase I hypersensitivity sites) and five histone modifications, including H3K4me3, H3K9me3, H3K27ac, H3K36me3, and H3K27me3, from the ENCODE project [53] (accession numbers in Supporting Information S1: Table S9). The number of peaks and the maximal signal intensity within each lncRNA gene were calculated as features. The final raw feature for an lncRNA was a 20‐dimensional vector.

The raw features for proteins represent the corresponding gene’s expression level, evolutionary conservation and functional complexity. For each tissue, we obtained the protein expression level from the ENCODE project [53] (accession numbers in Supporting Information S1: Table S9). We calculated the number of one‐to‐one orthologs across species based on the Ensembl database records [54]. This number was used to measure the evolutionary conservation of every protein. We obtained GO annotations for human and mouse proteins from the Ensembl BioMart [44] and the MGI database [55], respectively. The number of GO terms was used to describe the functional complexity of a gene. The final raw feature for a protein was a 3‐dimensional vector.

If a node in the LPPI networks did not have any raw feature, this node was removed from the LPPI network, along with all corresponding interactions. We applied a *z*‐score normalization before the HinSAGE module generated universal embeddings.

### 5.4 Raw feature aggregation on LPPI networks

Raw features of lncRNAs and proteins have different dimensions and different semantics. It is difficult to further integrate them in a traditional machine learning model. To address this, we introduced HinSAGE [56], which is specifically designed to produce universal node representations in this context. HinSAGE is an extension of GraphSAGE [57]. GraphSAGE works only on homogeneous networks, whereas HinSAGE can operate on heterogeneous graphs.

We implemented a two‐layer HinSAGE model using the StellarGraph library [56], which is further trained with DGI (Deep Graph Infomax) [58] to generate universal embeddings on the LPPI networks. Let **h**_*i*_^(*k*)^ ∈ R^*d*(*k*)^ be the universal representation of the *i*th node at the *k*th layer in a *d*(*k*) dimensional latent space. We first generate the aggregation at the *k*th layer for type *t* neighbors of the *i*th node with sampling, as follows:

(1) $$h_{N_{t}\left(i\right)}^{\left(k\right)}=\frac{1}{\left|N_{t}\left(i\right)\right|}\sum_{u\in N_{t}\left(i\right)}h_{u}^{\left(k-1\right)},$$

where *N*_*t*_(*i*) denotes the set of type *t* neighbor samples for the *i*th node. The node representation is then updated by concatenating its self‐representation with the aggregation of neighbors, as follows:

(2) $$h_{i}^{\left(k\right)}=\sigma \left\{\text{CONCAT}\left[W_{\text{self},\tau \left(i\right)}^{\left(k\right)}h_{i}^{\left(k-1\right)},\frac{1}{\left|T\right|}\sum_{t\in T}W_{\text{neigh},t}^{\left(k\right)}h_{N_{t}\left(i\right)}^{\left(k\right)}\right]+b^{\left(k\right)}\right\},$$

where *T* is the set of all neighbor types for the *i*th node, *τ*(*i*), the type of the *i*th node, **b** ^(*k*)^ ∈ R^*d*(*k*)^, a bias, $W_{\text{self},\tau \left(i\right)}^{\left(k\right)}$ ∈ R^*d*(*k*)×*d*(*k*)/2^, the self‐connection weight matrix for the *i*th node, and $W_{\text{neigh},t}^{\left(k\right)}$ ∈ R^*d*(*k*)×*d*(*k*)/2^, the weight matrix for type *t* neighbors. Specifically, when *k* = 1, $W_{\text{self},\tau \left(i\right)}^{\left(1\right)}$ projects the raw features of lncRNAs (**h**_*i*_^(0)^ ∈ R^20^) and proteins (**h**_*i*_^(0)^ ∈ R^3^) into a shared latent space R^*d*(*k*)/2^.

### 5.5 LncRNA gene essentiality estimations

To train supervised machine learning models for finding essential lncRNAs, a high‐quality set of nonessential lncRNA genes is required. We applied GIC scores to prioritize lncRNAs without essentiality marks on the LPPI networks [17, 20]. Those with the lowest GIC scores were selected as the nonessential lncRNA genes. The size of the nonessential lncRNA gene set was the same as that of the essential lncRNA gene set, thereby constructing a balanced benchmarking dataset.

With the aggregated universal representations of lncRNA genes, we trained SVM and MLP models using the scikit‐learn library [59]. Due to different dataset sizes between the human and the mouse, we applied the 10‐fold cross‐validations on human tissues, while the leave‐one‐out cross‐validations on mouse tissues. The benchmarking performances were measured mainly using the AUROC and the AUPR. Additional performance measures (Sen, Spe, PPV, Acc, F_1_, and MCC) were also calculated. We applied our classifiers to screen the entire LPPI network in each tissue, producing comprehensive estimations of essentialities for all lncRNA genes.

### 5.6 Parameter calibrations

We used human left ventricle and mouse heart as examples for parameter calibrations. For the two‐layer HinSAGE model, we optimized the dimensions of the hidden layers (*d* (1), *d* (2)) and the number of sampled neighboring nodes (*n*_1_, *n*_2_) in this order. We scanned *d* (1) ∈ {16, 32, 64}, *d* (2) ∈ {32, 64, 128, 256}, *n*_1_ ∈ {5, 10, 15, 20}, and *n*_2_ ∈ {10, 15, 20, 25}. When optimizing the dimensions of the hidden layers, the number of neighboring nodes sampled was set to *n*_1_ = 10, *n*_2_ = 15. The optimized parameters were *n*_1_ = 5, *n*_2_ = 25, *d* (1) = 32, *d* (2) = 256 for the human and *n*_1_ = 10, *n*_2_ = 15, *d* (1) = 64, *d* (2) = 256 for the mouse.

We implemented the SVM with a radial basis function kernel. We performed a grid search for the regularization parameter *C* and kernel coefficient *γ*. The grid was composed of *C* ∈ {1, 10, 100} and *γ* ∈ {0.1, 0.01, 0.001}. The optimal value was *C* = 100 and *γ* = 0.001 for the human and *C* = 10 and *γ* = 0.01 for the mouse.

The structure of the MLP was designed with two hidden layers. The parameters that needed adjustment were the number of neurons in the two hidden layers (*m*_1_ and *m*_2_). We scanned *m*_1_, *m*_2_ ∈ {32, 64, 128, 256} for the human and the mouse. During hyperparameter optimization, we restricted the search space to nonincreasing layer architectures (*m*_2_ ≤ *m*_1_). The optimal values were *m*_1_ = 128, m_2_ = 64 for the human, and *m*_1_ = 256, m_2_ = 32 for the mouse. All parameters in the GIC were set according to its original report [17].

## AUTHOR CONTRIBUTIONS

**Wan‐Ting Shi**: Data curation; formal analysis; investigation; methodology; software; validation; visualization; writing—original draft. **Ying‐Dong Liu**: Software; visualization. **Xiu‐Jun Gong**: Conceptualization; investigation; supervision; writing—review and editing. **Pu‐Feng Du**: Conceptualization; funding acquisition; investigation; project administration; supervision; writing—original draft; writing—review and editing.

## CONFLICT OF INTEREST STATEMENT

The authors declare no conflicts of interest.

## ETHICS STATEMENT

This article does not involve any studies with human or animal subjects conducted by any of the authors.

## Supporting information

Supporting Information S1

## Data Availability

The code and data for reproducing the results of this paper are available in a GitHub repository (swt1024/ELE).
