# Supplementary material for: ELE: Estimating tissue‐specific long noncoding RNA gene essentiality using graph neural networks
Source: Quant Biol. 2026 Sep 21;14(4):e70053. doi: 10.1002/qub2.70053 (PMC13642078; doi:10.1002/qub2.70053)
Supplement: Supplementary file 1 [file QUB2-14-e70053-s001.pdf]

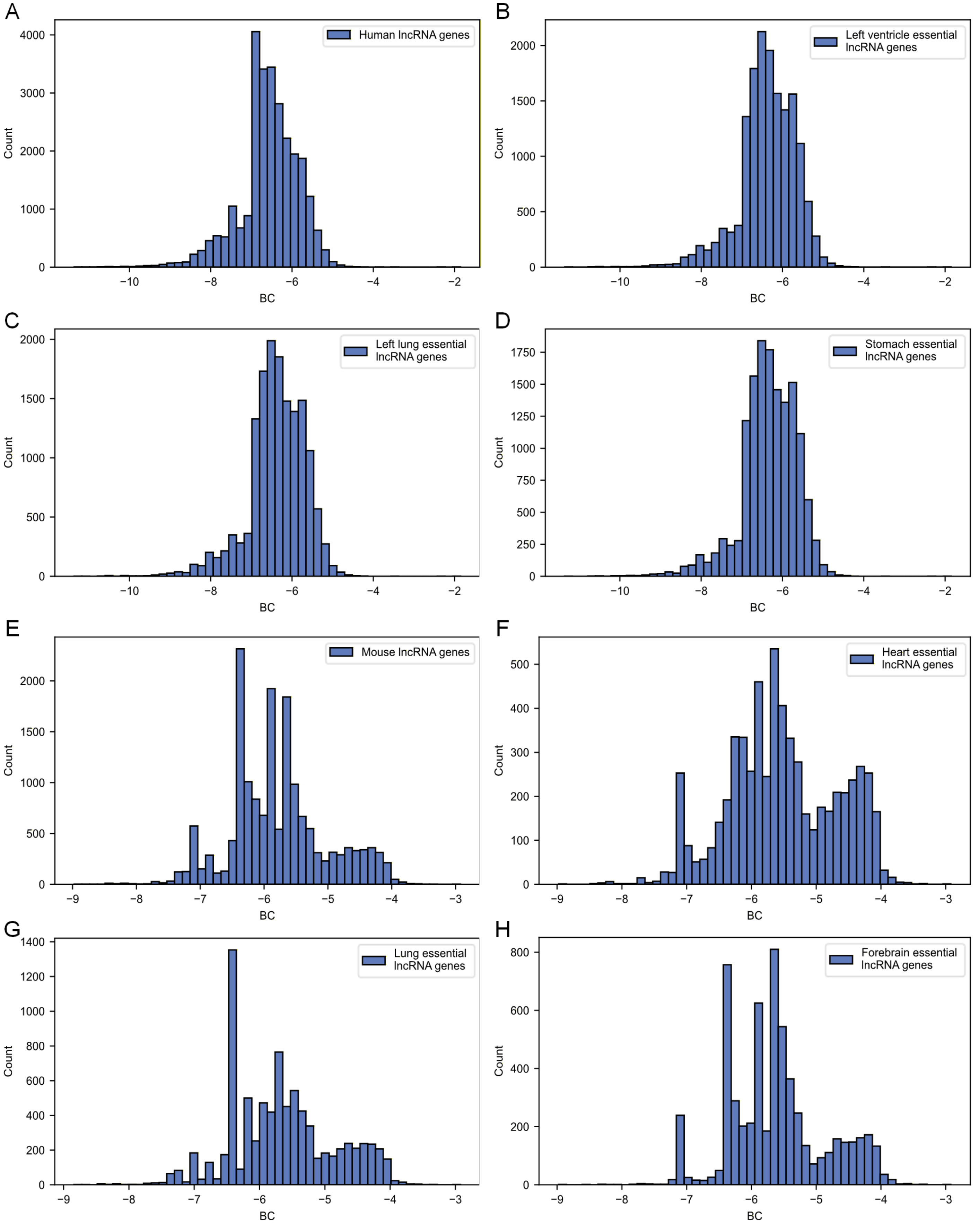

Figure S1 - BC (Betweenness Centrality) distribution among different organisms and tissues. (A) for all human lncRNA genes; (B) predicted essential lncRNA genes in left ventricle; (C) predicted essential lncRNA genes in left lung; (D) predicted essential lncRNA genes in stomach; (E) for all mouse lncRNA genes; (F) predicted essential lncRNA genes in heart; (G) predicted essential lncRNA genes in lung; (H) predicted essential lncRNA genes in forebrain.

Table S1 - Predictive performance of ELE

Classifier	Tissue	Sen	Spe	PPV	F1	ACC	MCC
SVM	Human left ventricle	81.78%	82.22%	82.14%	81.96%	82.00%	63.99%
SVM	Human left lung	80.32%	82.36%	81.99%	81.15%	81.34%	62.70%
SVM	Human stomach	80.39%	81.12%	80.98%	80.69%	80.76%	61.52%
SVM	Mouse heart	87.50%	91.67%	91.30%	89.36%	89.58%	79.24%
SVM	Mouse lung	75.00%	91.67%	90.00%	81.82%	83.33%	67.61%
SVM	Mouse forebrain	70.83%	83.33%	80.95%	75.56%	77.08%	54.59%
MLP	Human left ventricle	78.06%	81.85%	81.14%	79.57%	79.96%	59.96%
MLP	Human left lung	79.15%	82.00%	81.47%	80.30%	80.58%	61.18%
MLP	Human stomach	78.13%	79.08%	78.88%	78.51%	78.61%	57.22%
MLP	Mouse heart	91.67%	91.67%	91.67%	91.67%	91.67%	83.33%
MLP	Mouse lung	91.67%	91.67%	91.67%	91.67%	91.67%	83.33%
MLP	Mouse forebrain	83.33%	75.00%	76.92%	80.00%	79.17%	58.54%

Table S2 - Number of essential lncRNA genes and BC thresholds

Tissue	The number of predicted essential lncRNA genes	Thresholds
Human left ventricle	6727	5.84×10^{-7}
Human left lung	6471	5.81×10^{-7}
Human stomach	6063	6.54×10^{-7}
Mouse heart	4154	1.27×10^{-6}
Mouse lung	6202	5.86×10^{-7}
Mouse forebrain	4243	1.12×10^{-6}

Table S3 - GO enrichment of tissue-specific essential lncRNA genes

Species	Tissue	Category	Term	P-Value	Fold Enrichment	FDR
Human	left ventricle	GOTERM_BP_DIRECT	RNA processing	6.52E-29	2.63	3.33E-25
Human	left ventricle	GOTERM_BP_DIRECT	ubiquitin-dependent protein catabolic process	0.00000006	2.2	0.000153
Human	left ventricle	GOTERM_BP_DIRECT	chromatin remodeling	0.000000174	2.01	0.000297
Human	left ventricle	GOTERM_BP_DIRECT	regulation of G1/S transition of mitotic cell cycle	0.00000465	3.42	0.00517
Human	left ventricle	GOTERM_BP_DIRECT	regulation of cell cycle	0.00000505	1.97	0.00517
Human	left ventricle	GOTERM_CC_DIRECT	nucleolus	4.58E-29	1.99	4.4E-26
Human	left ventricle	GOTERM_CC_DIRECT	nucleoplasm	8.11E-24	1.46	3.89E-21
Human	left ventricle	GOTERM_CC_DIRECT	cytosol	1.26E-23	1.37	4.02E-21
Human	left ventricle	GOTERM_CC_DIRECT	cytoplasm	3.97E-22	1.28	9.52E-20
Human	left ventricle	GOTERM_CC_DIRECT	nucleus	8.64E-22	1.29	1.66E-19
Human	left ventricle	GOTERM_MF_DIRECT	protein binding	4.73E-32	1.16	7.36E-29
Human	left ventricle	GOTERM_MF_DIRECT	RNA binding	1.59E-19	1.74	1.23E-16
Human	left ventricle	GOTERM_MF_DIRECT	cadherin binding	5.86E-10	2.27	0.000000303
Human	left ventricle	GOTERM_MF_DIRECT	mRNA binding	7.05E-09	2.3	0.00000274
Human	left ventricle	GOTERM_MF_DIRECT	nucleic acid binding	0.000000374	1.69	0.000116
Human	left lung	GOTERM_BP_DIRECT	RNA processing	3.69E-35	2.89	1.86E-31
Human	left lung	GOTERM_BP_DIRECT	translation	0.000000651	1.99	0.00112
Human	left lung	GOTERM_BP_DIRECT	cytoplasmic translation	0.000000756	2.7	0.00112
Human	left lung	GOTERM_BP_DIRECT	chromatin remodeling	0.000000891	1.97	0.00112
Human	left lung	GOTERM_BP_DIRECT	rhythmic process	0.00000343	2.44	0.00345
Human	left lung	GOTERM_CC_DIRECT	nucleolus	3.69E-34	2.12	3.34E-31
Human	left lung	GOTERM_CC_DIRECT	cytosol	7.8E-23	1.37	3.53E-20
Human	left lung	GOTERM_CC_DIRECT	cytoplasm	1.95E-20	1.27	5.88E-18
Human	left lung	GOTERM_CC_DIRECT	nucleoplasm	7.43E-20	1.43	1.68E-17
Human	left lung	GOTERM_CC_DIRECT	nucleus	1.04E-18	1.28	1.88E-16
Human	left lung	GOTERM_MF_DIRECT	protein binding	1.01E-30	1.16	1.52E-27
Human	left lung	GOTERM_MF_DIRECT	RNA binding	1.69E-19	1.77	1.27E-16
Human	left lung	GOTERM_MF_DIRECT	mRNA binding	9.99E-10	2.43	0.000000501
Human	left lung	GOTERM_MF_DIRECT	cadherin binding	3.32E-09	2.25	0.00000125
Human	left lung	GOTERM_MF_DIRECT	nucleic acid binding	0.00000042	1.71	0.000126
Human	stomach	GOTERM_BP_DIRECT	RNA processing	1.1E-34	3.03	5.16E-31

Species	Tissue	Category	Term	P-Value	Fold Enrichment	FDR
Human	stomach	GOTERM_CC_DIRECT	nucleolus	2.57E-29	2.11	2.26E-26
Human	stomach	GOTERM_CC_DIRECT	cytoplasm	2.11E-18	1.27	9.27E-16
Human	stomach	GOTERM_CC_DIRECT	nucleus	1.59E-16	1.28	3.87E-14
Human	stomach	GOTERM_CC_DIRECT	cytosol	1.76E-16	1.33	3.87E-14
Human	stomach	GOTERM_CC_DIRECT	nucleoplasm	1.13E-13	1.37	1.99E-11
Human	stomach	GOTERM_MF_DIRECT	protein binding	7.31E-24	1.15	1.04E-20
Human	stomach	GOTERM_MF_DIRECT	RNA binding	2.47E-15	1.73	1.75E-12
Human	stomach	GOTERM_MF_DIRECT	cadherin binding	2.79E-08	2.27	0.0000132
Human	stomach	GOTERM_MF_DIRECT	mRNA binding	5.01E-08	2.37	0.0000178
Human	stomach	GOTERM_MF_DIRECT	nucleic acid binding	0.0000257	1.63	0.0073
Human	common	GOTERM_BP_DIRECT	RNA processing	1.32E-31	3.25	5.38E-28
Human	common	GOTERM_CC_DIRECT	nucleolus	1.82E-26	2.2	1.46E-23
Human	common	GOTERM_CC_DIRECT	cytoplasm	6.61E-13	1.26	2.65E-10
Human	common	GOTERM_CC_DIRECT	nucleoplasm	3.77E-12	1.4	1.01E-09
Human	common	GOTERM_CC_DIRECT	nucleus	1.73E-11	1.26	3.47E-09
Human	common	GOTERM_CC_DIRECT	cytosol	3.86E-11	1.3	6.19E-09
Human	common	GOTERM_MF_DIRECT	protein binding	1.2E-17	1.15	1.53E-14
Human	common	GOTERM_MF_DIRECT	RNA binding	5.27E-12	1.72	3.34E-09
Human	common	GOTERM_MF_DIRECT	mRNA binding	0.00000141	2.39	0.000545
Human	common	GOTERM_MF_DIRECT	cadherin binding	0.00000172	2.25	0.000545
Human	common	GOTERM_MF_DIRECT	nucleic acid binding	0.000025	1.73	0.00634
Mouse	heart	GOTERM_BP_DIRECT	endocytosis	1.16E-13	3.18	5.91E-10
Mouse	heart	GOTERM_BP_DIRECT	chromatin organization	6.15E-13	2.41	1.56E-09
Mouse	heart	GOTERM_BP_DIRECT	mRNA processing	2.31E-12	2.41	3.92E-09
Mouse	heart	GOTERM_BP_DIRECT	protein stabilization	1.96E-11	2.91	2.49E-08
Mouse	heart	GOTERM_BP_DIRECT	in utero embryonic development	4.64E-11	2.44	4.72E-08
Mouse	heart	GOTERM_CC_DIRECT	cytoplasm	2.88E-38	1.45	2.56E-35
Mouse	heart	GOTERM_CC_DIRECT	nucleus	2.19E-36	1.49	9.73E-34
Mouse	heart	GOTERM_CC_DIRECT	cytosol	4.29E-36	1.64	1.27E-33
Mouse	heart	GOTERM_CC_DIRECT	glutamatergic synapse	2.18E-28	2.47	4.84E-26
Mouse	heart	GOTERM_CC_DIRECT	nucleoplasm	3.5E-25	1.59	6.22E-23
Mouse	heart	GOTERM_MF_DIRECT	protein binding	8.86E-44	1.58	1.34E-40

Species	Tissue	Category	Term	P-Value	Fold Enrichment	FDR
Mouse	heart	GOTERM_MF_DIRECT	RNA binding	2.46E-16	1.96	1.86E-13
Mouse	heart	GOTERM_MF_DIRECT	enzyme binding	1.87E-12	2.25	9.42E-10
Mouse	heart	GOTERM_MF_DIRECT	protein-containing complex binding	7.38E-12	1.99	2.78E-09
Mouse	heart	GOTERM_MF_DIRECT	ubiquitin protein ligase binding	3.59E-11	2.44	1.08E-08
Mouse	lung	GOTERM_BP_DIRECT	positive regulation of transcription by RNA polymerase II	6.34E-20	1.68	3.43E-16
Mouse	lung	GOTERM_BP_DIRECT	chromatin organization	1.19E-19	2.27	3.43E-16
Mouse	lung	GOTERM_BP_DIRECT	protein ubiquitination	1.55E-19	2.13	3.43E-16
Mouse	lung	GOTERM_BP_DIRECT	mRNA processing	5.52E-19	2.29	9.15E-16
Mouse	lung	GOTERM_BP_DIRECT	positive regulation of DNA-templated transcription	7.02E-19	1.89	9.32E-16
Mouse	lung	GOTERM_CC_DIRECT	cytoplasm	2E-100	1.56	2.18E-97
Mouse	lung	GOTERM_CC_DIRECT	nucleus	7.42E-94	1.59	4.05E-91
Mouse	lung	GOTERM_CC_DIRECT	cytosol	3.06E-77	1.71	1.11E-74
Mouse	lung	GOTERM_CC_DIRECT	nucleoplasm	4.23E-55	1.67	1.15E-52
Mouse	lung	GOTERM_CC_DIRECT	glutamatergic synapse	8.07E-39	2.26	1.76E-36
Mouse	lung	GOTERM_MF_DIRECT	protein binding	3.56E-82	1.59	7.25E-79
Mouse	lung	GOTERM_MF_DIRECT	zinc ion binding	2.08E-22	1.54	2.11E-19
Mouse	lung	GOTERM_MF_DIRECT	metal ion binding	4.84E-22	1.36	3.28E-19
Mouse	lung	GOTERM_MF_DIRECT	protein kinase binding	8.8E-22	2.13	4.47E-19
Mouse	lung	GOTERM_MF_DIRECT	transferase activity	8.59E-21	1.54	3.5E-18
Mouse	forebrain	GOTERM_BP_DIRECT	chromatin organization	1.19E-22	2.67	7.23E-19
Mouse	forebrain	GOTERM_BP_DIRECT	protein ubiquitination	1.59E-18	2.31	4.84E-15
Mouse	forebrain	GOTERM_BP_DIRECT	positive regulation of transcription by RNA polymerase II	7.99E-18	1.76	1.62E-14
Mouse	forebrain	GOTERM_BP_DIRECT	positive regulation of DNA-templated transcription	2.01E-17	2.01	3.07E-14
Mouse	forebrain	GOTERM_BP_DIRECT	mRNA processing	4.93E-17	2.45	6.01E-14
Mouse	forebrain	GOTERM_CC_DIRECT	cytoplasm	8.47E-92	1.62	8.5E-89
Mouse	forebrain	GOTERM_CC_DIRECT	nucleus	2.63E-85	1.66	1.32E-82
Mouse	forebrain	GOTERM_CC_DIRECT	cytosol	2.27E-66	1.77	7.61E-64
Mouse	forebrain	GOTERM_CC_DIRECT	nucleoplasm	4.4E-53	1.77	1.11E-50
Mouse	forebrain	GOTERM_CC_DIRECT	glutamatergic synapse	1.09E-34	2.41	2.19E-32
Mouse	forebrain	GOTERM_MF_DIRECT	protein binding	7.31E-76	1.66	1.35E-72
Mouse	forebrain	GOTERM_MF_DIRECT	transferase activity	1.01E-23	1.7	9.36E-21
Mouse	forebrain	GOTERM_MF_DIRECT	metal ion binding	1.61E-23	1.43	9.9E-21

Species	Tissue	Category	Term	P-Value	Fold Enrichment	FDR
Mouse	forebrain	GOTERM_MF_DIRECT	RNA binding	4.95E-23	2	2.28E-20
Mouse	forebrain	GOTERM_MF_DIRECT	nucleotide binding	1.25E-20	1.65	4.34E-18
Mouse	common	GOTERM_BP_DIRECT	intracellular signal transduction	1.64E-10	2.93	0.000000618
Mouse	common	GOTERM_BP_DIRECT	chromatin organization	4.74E-10	2.92	0.000000894
Mouse	common	GOTERM_BP_DIRECT	negative regulation of transcription by RNA polymerase II	1.53E-08	2.04	0.0000193
Mouse	common	GOTERM_BP_DIRECT	mRNA processing	2.94E-08	2.74	0.0000277
Mouse	common	GOTERM_BP_DIRECT	DNA damage response	5.85E-08	2.41	0.0000442
Mouse	common	GOTERM_CC_DIRECT	cytoplasm	2.23E-41	1.72	1.49E-38
Mouse	common	GOTERM_CC_DIRECT	cytosol	3.72E-36	1.99	1.24E-33
Mouse	common	GOTERM_CC_DIRECT	nucleus	9.49E-29	1.65	2.11E-26
Mouse	common	GOTERM_CC_DIRECT	nucleoplasm	1.17E-17	1.74	1.96E-15
Mouse	common	GOTERM_CC_DIRECT	glutamatergic synapse	9.93E-12	2.36	1.33E-09
Mouse	common	GOTERM_MF_DIRECT	protein binding	3.28E-36	1.79	3.44E-33
Mouse	common	GOTERM_MF_DIRECT	protein-containing complex binding	1.44E-12	2.64	7.53E-10
Mouse	common	GOTERM_MF_DIRECT	transferase activity	3.59E-10	1.77	8.92E-08
Mouse	common	GOTERM_MF_DIRECT	protein serine/threonine kinase activity	3.94E-10	2.85	8.92E-08
Mouse	common	GOTERM_MF_DIRECT	protein serine kinase activity	4.25E-10	3.07	8.92E-08

Table S4 - Statistics of lncRNA genes interacting with essential proteins

Species	LncRNA gene	Interacting with essential proteins	Interacting with non-essential proteins
Human	Essential in left ventricle	6709	18
Human	Essential in left lung	6451	20
Human	Essential in stomach	6050	13
Human	All lncRNA genes	25994	9338
Mouse	Essential in heart	3902	252
Mouse	Essential in lung	5759	443
Mouse	Essential in forebrain	4153	90
Mouse	All lncRNA genes	23798	5017

Table S5 - Statistics of the LPPI network in ELE

Genome	Human	Mouse
LncRNA genes	35332	28815
Proteins	17125	11200
LncRNA-protein interactions	438057	107023
Protein-protein interactions	794780	67226
Total lncRNA samples	2744	48
Essential lncRNA genes (Positive)	1372	24
Non-essential lncRNA genes (Negative)	1372	24

Table S6 - Human essential lncRNA genes in the benchmarking dataset

Unified node ID	Genomic coordinates	Matched evidence ID[1]
l000005	chr1:89294-297502:-	NONHSAG000008
l000022	chr1:817370-859572:+	NONHSAG000047
l000026	chr1:914887-925614:-	LINC02593;NONHSAG000059;RP11-54O7.3
l000034	chr1:1020119-1056119:+	NONHSAG000069
l000072	chr1:1659026-1662939:+	NONHSAG056053
l000075	chr1:1702378-1745992:-	RP1-283E3.8
l000079	chr1:1891174-1897138:+	NONHSAG000130
l000099	chr1:2492299-2493258:-	NONHSAG000158
l000129	chr1:3734285-3747375:-	NONHSAG000195
l000165	chr1:6443033-6447006:-	NONHSAG000241
l000173	chr1:6724635-6730013:+	NONHSAG000253
l000208	chr1:9139554-9202826:-	MIR34AHG;NONHSAG000310
l000209	chr1:9181549-9198866:+	LNCTAM34A;NONHSAG000311
l000250	chr1:11609530-11613356:+	NONHSAG000374
l000257	chr1:11777076-11779619:-	NONHSAG000381
l000309	chr1:16155071-16188290:+	NONHSAG000464;RP11-276H7.2
l000322	chr1:16618257-16645479:-	NONHSAG000484
l000329	chr1:16740272-16972964:+	NONHSAG000498;RP11-108M9.3
l000387	chr1:20642656-20652193:-	NONHSAG000579
l000391	chr1:20732248-20733953:+	NONHSAG000584
l000404	chr1:21440049-21455432:+	NONHSAG000603
l000414	chr1:22023989-22031214:-	NONHSAG000625
l000491	chr1:25816586-25823606:-	NONHSAG000742

Unified node ID	Genomic coordinates	Matched evidence ID[1]
l000507	chr1:26429547-26473892:-	NONHSAG000769
l000562	chr1:28573544-28582983:-	NONHSAG000840
l000564	chr1:28643158-28651098:+	LINC01715;NONHSAG000843
l000581	chr1:29708850-29709548:-	NONHSAG000863
l000595	chr1:30858157-30860254:+	NONHSAG000884
l000618	chr1:31789129-31791323:+	NONHSAG000918
l000631	chr1:32231659-32241710:-	NONHSAG000938
l000650	chr1:32986951-33058910:+	NONHSAG000967
l000778	chr1:40133032-40135704:+	NONHSAG001142
l000803	chr1:40863047-40876671:+	NONHSAG001172
l000838	chr1:42958276-43196506:+	NONHSAG058967;SLC2A1-AS1
l000852	chr1:43453883-43457111:+	NONHSAG001238
l000859	chr1:43937015-43946659:-	NONHSAG001247
l000867	chr1:44030413-44115937:+	NONHSAG001259
l000881	chr1:44759036-44775846:-	NONHSAG001278
l000885	chr1:44816602-44819945:+	NONHSAG001284
l000934	chr1:47096652-47181087:-	CYP4A22-AS1;NONHSAG001360
l000940	chr1:47380714-47437695:-	NONHSAG001375
l000984	chr1:51329628-51354342:+	NONHSAG001448
l000996	chr1:52050917-52052683:+	NONHSAG001468
l001004	chr1:52353486-52353877:+	NONHSAG056451
l001016	chr1:53209782-53213776:-	NONHSAG001499
l001030	chr1:53348488-53350306:-	NONHSAG001513
l001139	chr1:63319649-63323742:-	NONHSAG001696
l001157	chr1:64833222-65067754:-	NONHSAG001724

Unified node ID	Genomic coordinates	Matched evidence ID[1]
l001185	chr1:68385473-68484266:+	NONHSAG001773
l001231	chr1:74341578-74378802:-	NONHSAG001837
l001294	chr1:84074516-84078555:-	NONHSAG001941
l001338	chr1:87129764-87169204:+	NONHSAG001993
l001385	chr1:91682716-91712276:-	NONHSAG002056
l001386	chr1:91796909-91886107:-	NONHSAG002058
l001408	chr1:93261701-93346434:-	NONHSAG002086
l001432	chr1:94926001-95067546:+	NONHSAG002135
l001433	chr1:94982722-95072951:-	NONHSAG002134
l001521	chr1:105927619-106028277:-	LINC01677;NONHSAG058006;RP11-24P14.1
l001564	chr1:109539905-109548563:-	NONHSAG002343;RP5-1160K1.8
l001568	chr1:109668021-109785336:+	NONHSAG056733
l001573	chr1:109884099-109897700:-	NONHSAG002359
l001583	chr1:110282598-110339216:-	NONHSAG002376;RBM15-AS1;RP5-1074L1.1
l001585	chr1:110347053-110443817:+	LAMTOR5-AS1;NONHSAG002378
l001596	chr1:110963301-110964649:+	NONHSAG056746
l001625	chr1:111989533-112008406:+	LINC01750
l001640	chr1:112849769-112885214:+	LINC01357;NONHSAG002454
l001664	chr1:114399558-114427782:-	NONHSAG002490
l001701	chr1:117295465-117321337:+	NONHSAG002549
l001738	chr1:120912237-121009291:+	NONHSAG002669
l001747	chr1:121396239-121463508:+	LINC02798;NONHSAG002617
l001792	chr1:145926477-145959179:+	LIX1L-AS1;NONHSAG002701
l001812	chr1:147258884-147517875:-	NONHSAG002731
l001818	chr1:148009420-148014956:-	NONHSAG058109

Unified node ID	Genomic coordinates	Matched evidence ID[1]
l001819	chr1:148011347-148025934:+	LINC02804
l001821	chr1:148156138-148186980:+	LINC02805;NONHSAG002752
l001822	chr1:148159212-148263380:-	NONHSAG002719
l001828	chr1:148290888-148519604:-	NONHSAG058112;RP11-495P10.8
l001845	chr1:149722925-149727147:-	NONHSAG058118
l001865	chr1:150255094-150259933:-	RP4-790G17.7
l001876	chr1:150548561-150558474:-	NONHSAG002859
l001878	chr1:150560201-150574552:-	NONHSAG058128
l001892	chr1:150971915-150981215:+	NONHSAG002883
l001953	chr1:153618802-153634397:-	NONHSAG002978
l001982	chr1:154480011-154481501:+	NONHSAG056868
l002010	chr1:155194805-155207358:+	NONHSAG003053
l002012	chr1:155211150-155213819:-	NONHSAG003056
l002023	chr1:155312884-155324385:-	NONHSAG003068;RUSC1-AS1
l002026	chr1:155561980-155614951:+	NONHSAG003074
l002028	chr1:155609338-155610380:-	NONHSAG003077
l002032	chr1:155710097-155710563:-	RP11-243J18.2
l002040	chr1:155978634-155983436:+	NONHSAG056875
l002059	chr1:156388092-156395609:+	NONHSAG003119
l002072	chr1:156711366-156713190:-	NONHSAG003134
l002085	chr1:157526624-157697947:+	NONHSAG003156
l002108	chr1:159961174-160022889:+	NONHSAG003198
l002139	chr1:161140943-161159418:-	NONHSAG003244
l002152	chr1:161323613-161363187:+	NONHSAG003260
l002157	chr1:161505429-161605662:+	NONHSAG003269

Unified node ID	Genomic coordinates	Matched evidence ID[1]
l002183	chr1:163244471-163321894:-	NONHSAG003303
l002224	chr1:167425026-167518640:-	NONHSAG003374
l002299	chr1:173851284-173869006:-	NONHSAG003504
l002300	chr1:173862151-173863942:+	GAS5-AS1;NONHSAG003505
l002303	chr1:173931083-174022357:-	NONHSAG003512;RC3H1-IT1
l002398	chr1:181174483-181182208:+	NONHSAG003645
l002468	chr1:186680594-186684913:+	NONHSAG057043
l002502	chr1:193121982-193254815:+	NONHSAG003820
l002534	chr1:198638456-198759342:+	NONHSAG003874
l002536	chr1:198739202-199021038:-	MIR181A1HG;NONHSAG059768
l002539	chr1:198898729-198906565:+	NONHSAG003884
l002541	chr1:198915729-198916300:+	NONHSAG003886
l002549	chr1:200323811-200483546:+	NONHSAG003900
l002557	chr1:201023868-201043642:+	NONHSAG003911
l002558	chr1:201070260-201074573:-	NONHSAG003912
l002570	chr1:201688255-201829559:-	NONHSAG003938
l002582	chr1:202331676-202333478:-	NONHSAG003957
l002591	chr1:202810881-202814455:+	NONHSAG003976
l002619	chr1:204131061-204131966:+	NONHSAG004013
l002624	chr1:204218852-204378303:-	NONHSAG004026
l002643	chr1:205434884-205457091:-	NONHSAG004054
l002670	chr1:206634132-206636239:-	NONHSAG004095
l002772	chr1:212851961-212858309:-	FLVCR1-DT;NONHSAG004247
l002804	chr1:218344159-218346129:-	NONHSAG004303
l002805	chr1:218442625-218443996:+	NONHSAG057222

Unified node ID	Genomic coordinates	Matched evidence ID[1]
l002806	chr1:218459264-218525986:+	NONHSAG004305
l002837	chr1:221791088-222064817:-	NONHSAG004373
l002865	chr1:223992729-224034927:+	NONHSAG057260
l002967	chr1:228995678-229271599:-	NONHSAG058533;RP5-1061H20.4
l003007	chr1:231519610-231528751:-	NONHSAG004619
l003041	chr1:234357005-234370332:+	NONHSAG004673
l003042	chr1:234365244-234373857:-	COA6-AS1;NONHSAG004674
l003074	chr1:235367359-235452443:+	NONHSAG004714
l003090	chr1:236523068-236524531:-	NONHSAG004743
l003110	chr1:239898015-239899873:-	NONHSAG004779
l003167	chr1:244840242-244864560:-	HNRNPU-AS1;NONHSAG004868
l003250	chr2:677118-699331:+	NONHSAG026781
l003257	chr2:900215-905452:-	NONHSAG026794
l003294	chr2:3481241-3482410:-	NONHSAG026852
l003296	chr2:3531812-3536980:-	NONHSAG026854
l003298	chr2:3558095-3571313:+	NONHSAG026856
l003411	chr2:11677594-11827409:+	NONHSAG027017
l003442	chr2:15918349-15943052:-	NONHSAG027075
l003462	chr2:17797564-17800158:-	NONHSAG027110
l003567	chr2:26194218-26196447:+	NONHSAG027276
l003568	chr2:26213064-26215013:+	NONHSAG027278
l003601	chr2:27356245-27367881:+	NONHSAG027323
l003619	chr2:27889455-27891114:-	NONHSAG027342
l003642	chr2:29319321-29358220:+	NONHSAG027374
l003671	chr2:32377630-32379599:-	BIRC6-AS1;NONHSAG027421

Unified node ID	Genomic coordinates	Matched evidence ID[1]
l003704	chr2:37324895-37339716:+	NONHSAG027482
l003769	chr2:42958959-43027784:-	AC016735.1;NONHSAG027598
l003782	chr2:43995984-43996754:+	NONHSAG027618
l003819	chr2:46811582-46859007:+	NONHSAG027675
l003827	chr2:47067452-47198470:-	NONHSAG027682
l003830	chr2:47325857-47344517:+	BCYRN1
l003832	chr2:47412532-47429883:+	NONHSAG027694
l003842	chr2:47904970-47920233:+	NONHSAG027709
l003921	chr2:58159242-58241410:-	NONHSAG027837
l003944	chr2:61140657-61178345:-	NONHSAG027874
l003945	chr2:61177448-61188925:+	NONHSAG027876
l004026	chr2:67123356-67398136:-	NONHSAG028045
l004055	chr2:69700191-69713847:-	NONHSAG028083
l004060	chr2:69962262-70103221:-	NONHSAG028093
l004069	chr2:70301360-70303319:+	NONHSAG028112
l004091	chr2:71082536-71086300:+	NONHSAG028141
l004092	chr2:71110764-71130220:-	NONHSAG028142
l004102	chr2:72143678-72145788:+	NONHSAG028159
l004154	chr2:74498056-74507437:+	LBX2-AS1;NONHSAG077475
l004267	chr2:85815129-85826788:+	NONHSAG028403
l004289	chr2:87449517-87767359:+	NONHSAG077527
l004361	chr2:96208406-96254237:+	NONHSAG077551
l004423	chr2:100669891-100677586:-	LINC01868;NONHSAG028713
l004425	chr2:100736709-100747391:+	AC092168.3
l004428	chr2:100978453-101002245:-	NONHSAG028720

Unified node ID	Genomic coordinates	Matched evidence ID[1]
l004485	chr2:105324209-105330530:+	NONHSAG028817
l004487	chr2:105363037-105378842:+	NONHSAG028820
l004511	chr2:107382400-107748270:-	LINC01885;NONHSAG028857
l004517	chr2:108162553-108288736:-	NONHSAG028870
l004540	chr2:110231032-110287506:+	NONHSAG028913
l004541	chr2:110375106-110384951:-	NONHSAG028918
l004553	chr2:111145783-111699033:-	NONHSAG056025
l004557	chr2:111468809-111637967:+	NONHSAG028948
l004589	chr2:113099314-113134016:+	NONHSAG028996
l004611	chr2:113760276-113891064:-	NONHSAG029033
l004635	chr2:118124350-118224018:-	NONHSAG029080
l004665	chr2:121081436-121089204:-	AC067960.1;NONHSAG029124
l004743	chr2:130530274-130537182:+	NONHSAG029267
l004765	chr2:131630976-131649616:+	NONHSAG029312
l004767	chr2:131829800-131832031:-	AC103564.7
l004779	chr2:132285794-132294377:-	NONHSAG029336
l004780	chr2:132345615-132347356:-	AC097532.2
l004812	chr2:135985148-136022593:+	NONHSAG029377
l004855	chr2:144518096-144521491:+	NONHSAG029467
l004977	chr2:161222781-161308389:-	AC009299.3
l004978	chr2:161244737-161255442:+	NONHSAG029668
l005045	chr2:170618627-170818040:-	NONHSAG029792
l005073	chr2:172427773-172428604:-	NONHSAG029830
l005074	chr2:172464228-172556596:-	NONHSAG079231
l005103	chr2:174546996-174847427:+	NONHSAG029873

Unified node ID	Genomic coordinates	Matched evidence ID[1]
l005105	chr2:174757642-174759602:-	NONHSAG029880
l005129	chr2:176611019-176638186:-	LINC01116;NONHSAG080772
l005140	chr2:177263708-177267010:+	NONHSAG029933
l005155	chr2:178521182-178779963:+	NONHSAG029956;NONHSAG029957;NONHSAG029958;NONHSAG029959;NONHSAG029960;NONHSAG029961;NONHSAG029962;NONHSAG029965
l005156	chr2:178641271-178696072:-	NONHSAG029964
l005173	chr2:180678001-180921247:+	NONHSAG029985
l005241	chr2:189057317-189080019:-	NONHSAG030074
l005270	chr2:191209190-191246251:-	NONHSAG030113
l005281	chr2:192749802-192783952:+	NONHSAG030129;PCGEM1
l005365	chr2:201233442-201361836:+	NONHSAG030244
l005374	chr2:201620183-201643570:-	NONHSAG030258
l005378	chr2:201970944-202033544:-	NONHSAG110420
l005391	chr2:202374642-202376944:-	NONHSAG030278
l005392	chr2:202376326-202567751:+	NONHSAG030279
l005406	chr2:203328458-203329226:-	NONHSAG030304
l005424	chr2:206084550-206100326:+	NONHSAG030333
l005478	chr2:210477681-210679107:+	NONHSAG030410
l005496	chr2:214809182-214969553:+	NONHSAG030440;SNHG31
l005523	chr2:216694403-217336120:+	NONHSAG030487
l005536	chr2:218214013-218217078:-	NONHSAG030516
l005542	chr2:218363911-218399644:-	NONHSAG030525
l005575	chr2:219418747-219426737:+	NONHSAG030583
l005576	chr2:219425070-219426184:-	NONHSAG030582

Unified node ID	Genomic coordinates	Matched evidence ID[1]
l005662	chr2:230440434-230513294:-	NONHSAG030739
l005663	chr2:230494445-230542903:+	NONHSAG030738
l005664	chr2:230520593-230589562:-	NONHSAG030742
l005752	chr2:236910770-237085841:-	NONHSAG081227
l005835	chr2:241351339-241353105:-	NONHSAG031025
l005837	chr2:241360870-241372749:-	AC005104.3
l005843	chr2:241543822-241559673:-	NONHSAG031035
l005897	chr3:4735983-4887293:-	EGOT;NONHSAG034316
l005957	chr3:10081320-10108231:-	NONHSAG034417
l005958	chr3:10141777-10153676:+	NONHSAG034418
l006011	chr3:14144636-14165978:+	NONHSAG034492
l006027	chr3:14877561-14949563:-	NONHSAG034508
l006038	chr3:15436170-15455940:-	EAF1-AS1;NONHSAG034527
l006135	chr3:30650419-30671815:+	NONHSAG034676
l006136	chr3:30695515-30699576:+	NONHSAG034678
l006220	chr3:38609760-38622442:-	NONHSAG034801
l006258	chr3:41220846-41225051:-	NONHSAG034845
l006259	chr3:41239183-41240443:+	NONHSAG034846
l006322	chr3:45676037-45689179:-	NONHSAG034935
l006352	chr3:47163978-47246601:+	NONHSAG034973
l006437	chr3:49899218-49925046:+	NONHSAG035096
l006441	chr3:50099601-50100988:-	NONHSAG035103
l006557	chr3:57755449-57930003:+	NONHSAG035262
l006578	chr3:59050097-59811310:+	NONHSAG035287
l006612	chr3:64684869-65113835:+	ADAMTS9-AS2;NONHSAG084701

Unified node ID	Genomic coordinates	Matched evidence ID[1]
l006641	chr3:69763811-69879383:-	NONHSAG035382
l006685	chr3:75672261-75690066:+	NONHSAG035456
l006735	chr3:93980219-94036736:+	NONHSAG035531
l006761	chr3:98522569-98525893:+	NONHSAG035568
l006795	chr3:101676429-101718569:+	NONHSAG084832
l006928	chr3:116709234-116723582:+	NONHSAG035806
l007020	chr3:123584353-123716399:+	MYLK-AS1;NONHSAG035914;NONHSAG035919
l007021	chr3:123610048-123884332:-	NONHSAG035916;NONHSAG035923
l007093	chr3:128488080-128541828:+	NONHSAG084937
l007113	chr3:129315146-129330722:+	NONHSAG036047
l007120	chr3:129436714-129440017:-	NONHSAG036054
l007158	chr3:132722341-132874223:+	NONHSAG036115
l007162	chr3:133333376-133491163:-	BFSP2-AS1;NONHSAG036120
l007253	chr3:141916527-141938547:-	ATP1B3-AS1;NONHSAG036255
l007258	chr3:142449006-142578733:-	NONHSAG036263
l007265	chr3:142960649-143060725:+	NONHSAG036273
l007346	chr3:151538965-151928176:-	NONHSAG036403
l007348	chr3:151770427-151784894:+	NONHSAG036407
l007352	chr3:152219240-152269626:-	NONHSAG036412
l007354	chr3:152329784-152331604:-	NONHSAG036413
l007480	chr3:169083498-169663775:-	NONHSAG036612
l007482	chr3:169764462-169765060:-	NONHSAG036615
l007518	chr3:172041045-172042974:-	NONHSAG036667
l007553	chr3:177025256-177050114:-	NONHSAG036718
l007599	chr3:180989235-181836880:+	NONHSAG036783

Unified node ID	Genomic coordinates	Matched evidence ID[1]
l007636	chr3:184074023-184155299:-	NONHSAG085975
l007658	chr3:184546713-184556919:-	NONHSAG036866
l007689	chr3:186780978-186784179:-	NONHSAG036922
l007695	chr3:186810879-186827552:-	NONHSAG036926
l007714	chr3:188148243-188154098:-	LPP-AS2;NONHSAG111667
l007762	chr3:194287323-194322826:-	NONHSAG037025
l007783	chr3:195542777-195550648:+	NONHSAG037061
l007787	chr3:195635099-195739964:+	LINC00969;NONHSAG037073
l007793	chr3:195836192-195860486:-	LINC01983;NONHSAG037080
l007795	chr3:195900985-195903417:+	AC124944.3
l007796	chr3:195908075-195913987:+	NONHSAG037082;NONHSAG037083;TNK2-AS1
l007804	chr3:196142531-196178101:+	NONHSAG037096
l007836	chr3:196999459-197004745:+	NONHSAG037137
l007839	chr3:197345719-197506986:-	NONHSAG037146
l007840	chr3:197456115-197467408:+	AC128709.3
l007846	chr3:197660564-197665775:-	NONHSAG037164
l007898	chr4:1024905-1026898:-	NONHSAG037241
l007958	chr4:3633028-3633976:+	NONHSAG037348
l008012	chr4:7754076-7778928:+	NONHSAG037420
l008096	chr4:16178938-16183120:+	NONHSAG037559
l008296	chr4:41748292-41872775:+	NONHSAG037827
l008344	chr4:47913805-47990737:+	NONHSAG037891
l008365	chr4:52712257-52720660:+	NONHSAG037936
l008376	chr4:53496399-53549579:+	NONHSAG037946
l008416	chr4:56448007-56530486:-	NONHSAG038001

Unified node ID	Genomic coordinates	Matched evidence ID[1]
l008430	chr4:57109735-57207459:+	IGFBP7-AS1;NONHSAG038024
l008458	chr4:64276472-64409340:-	NONHSAG038060
l008478	chr4:67701276-67792112:+	NONHSAG038091
l008522	chr4:73073375-73258798:-	NONHSAG038162
l008643	chr4:83455931-83470462:+	NONHSAG038343
l008644	chr4:83469023-83523348:-	NONHSAG038344
l008681	chr4:88268739-88284694:-	NONHSAG038397
l008757	chr4:99949976-100195099:+	NONHSAG038502
l008781	chr4:102825040-102887965:+	NONHSAG038530;UBE2D3-AS1
l008800	chr4:105168196-105352948:-	NONHSAG038561
l008810	chr4:105927059-105932723:-	NONHSAG038578
l008872	chr4:112646475-112706894:-	MIR302CHG;NONHSAG038663
l008878	chr4:112818031-113384221:+	NONHSAG038682
l008880	chr4:112973271-113071962:-	NONHSAG038675;NONHSAG038676
l008982	chr4:127043429-127077762:-	NONHSAG038836
l009108	chr4:143513471-143514837:-	NONHSAG088562
l009134	chr4:145642335-145650624:-	NONHSAG039037
l009135	chr4:145657251-145660034:+	NONHSAG039038
l009210	chr4:153684201-153702391:+	NONHSAG039147
l009312	chr4:168828918-168833085:-	NONHSAG039293
l009314	chr4:168894483-169010252:-	NONHSAG039295
l009334	chr4:170343087-170372311:+	LINC02512;NONHSAG039319
l009499	chr4:187527680-187672642:-	NONHSAG089857
l009529	chr5:92150-139863:+	CTD-2231H16.1
l009532	chr5:213897-218148:-	NONHSAG039617

Unified node ID	Genomic coordinates	Matched evidence ID[1]
l009534	chr5:254373-271517:-	NONHSAG039620
l009544	chr5:521251-527448:+	NONHSAG039634
l009545	chr5:571563-612231:-	NONHSAG039637
l009555	chr5:946283-957075:+	NONHSAG039654
l009564	chr5:1170641-1182901:-	NONHSAG039664
l009568	chr5:1345173-1352063:+	NONHSAG039669
l009632	chr5:6582135-6589232:+	NONHSAG039749
l009694	chr5:10323546-10354117:-	NONHSAG039872
l009753	chr5:14628323-14664604:-	CTD-2165H16.4;NONHSAG039951
l009890	chr5:35678248-35854374:-	NONHSAG040179
l009896	chr5:36194821-36219691:-	NONHSAG040188
l009924	chr5:38434413-38469466:-	NONHSAG040226
l009927	chr5:38692411-38881535:-	NONHSAG091943
l009934	chr5:39331772-39462300:-	NONHSAG040241
l009953	chr5:41868974-41880210:+	NONHSAG040269
l009983	chr5:43571320-43603954:-	NONHSAG040320
l009989	chr5:44495093-44844597:-	NONHSAG040329
l010055	chr5:55936142-55941727:+	NONHSAG040408
l010075	chr5:57395059-57534504:+	NONHSAG040444
l010100	chr5:60945228-61073191:+	NONHSAG040481
l010143	chr5:66143507-66144796:-	NONHSAG040542
l010145	chr5:66196288-66571973:+	NONHSAG040544;RP11-305P14.1
l010155	chr5:67463350-67490719:-	NONHSAG040560
l010174	chr5:69096374-69166958:-	NONHSAG040601
l010227	chr5:73213844-73295258:+	NONHSAG040692

Unified node ID	Genomic coordinates	Matched evidence ID[1]
l010229	chr5:73444826-73448777:-	NONHSAG040700
l010249	chr5:74640022-74722647:+	NONHSAG040729
l010287	chr5:78342364-78360514:-	NONHSAG040804
l010324	chr5:80654358-80876815:+	NONHSAG040859
l010331	chr5:81204083-81301569:-	NONHSAG040870
l010356	chr5:83509877-83581321:-	NONHSAG040909;VCAN-AS1
l010373	chr5:87268010-87268774:+	NONHSAG040945
l010383	chr5:88268849-88439490:+	NONHSAG040958
l010386	chr5:88499582-88691057:-	LINC00461;NONHSAG040969
l010389	chr5:88691547-88695492:-	NONHSAG040976
l010406	chr5:91285662-91314529:-	NONHSAG041002
l010416	chr5:92823934-92886872:+	NONHSAG041017
l010419	chr5:93019662-93261424:-	NONHSAG091116
l010421	chr5:93395507-93607053:-	NONHSAG041023
l010570	chr5:112160525-112164277:+	NONHSAG041241
l010574	chr5:112419288-112420976:+	NONHSAG041250
l010577	chr5:112707533-112775653:+	NONHSAG041256
l010578	chr5:112828834-112837844:+	NONHSAG041257
l010596	chr5:115262493-115265779:+	NONHSAG112396
l010732	chr5:128339386-128369342:+	NONHSAG041480
l010766	chr5:132603309-132605028:+	NONHSAG041528
l010767	chr5:132630588-132664273:-	NONHSAG041529
l010815	chr5:134874370-134901635:+	NONHSAG041592
l010866	chr5:138347026-138349642:-	NONHSAG041670
l010892	chr5:139628367-139649728:-	CXXC5-AS1;NONHSAG041707

Unified node ID	Genomic coordinates	Matched evidence ID[1]
l010900	chr5:140071311-140114769:-	MALINC1;NONHSAG041716
l010931	chr5:141096204-141268592:-	NONHSAG041758
l010965	chr5:142310426-142326455:-	NONHSAG091284
l010995	chr5:146099405-146120413:+	NONHSAG041844
l011021	chr5:148452836-148469020:+	NONHSAG041883
l011058	chr5:150475530-150486185:-	NONHSAG041938
l011073	chr5:151253051-151726300:+	NONHSAG041966
l011081	chr5:151765858-151771508:-	NONHSAG041981
l011113	chr5:157104787-157114900:+	NONHSAG042033
l011121	chr5:157682984-157690266:-	NONHSAG042050
l011128	chr5:158225351-158234377:-	NONHSAG042062
l011138	chr5:159208189-159249997:+	LINC01932;NONHSAG042080
l011171	chr5:163437568-163448199:+	NONHSAG042132
l011175	chr5:163482603-163494058:-	NONHSAG042138
l011232	chr5:171387115-171411810:+	NONHSAG042218
l011256	chr5:173144161-173145040:-	NONHSAG042257
l011267	chr5:173578969-173585069:+	NONHSAG042270
l011292	chr5:175906938-175958538:-	NONHSAG042306
l011293	chr5:175972600-175987361:+	NONHSAG042307
l011338	chr5:177612804-177672277:-	NONHSAG042370
l011376	chr5:179657761-179731641:+	NONHSAG042433
l011402	chr5:180824603-180861858:-	HEIH
l011403	chr5:180829953-180840133:+	NONHSAG042476
l011410	chr5:181186976-181193104:-	CTC-338M12.2;NONHSAG091452
l011411	chr5:181190941-181203117:+	NONHSAG042491

Unified node ID	Genomic coordinates	Matched evidence ID[1]
l011418	chr5:181246465-181276419:+	NONHSAG042500
l011478	chr6:1575183-1653965:+	NONHSAG092907
l011534	chr6:2985297-3024771:-	NONHSAG042722
l011536	chr6:2993064-3022800:+	NONHSAG042724
l011588	chr6:3925316-4021709:-	NONHSAG042836
l011598	chr6:4185478-4190029:-	NONHSAG042855
l011658	chr6:7539658-7543119:-	NONHSAG042957
l011663	chr6:8102710-8838822:+	NONHSAG042966;NONHSAG042970
l011675	chr6:10427784-10434875:-	NONHSAG042991
l011775	chr6:20400748-20403988:-	NONHSAG043138
l011788	chr6:21664219-22317798:+	NONHSAG056029
l011789	chr6:22124464-22222644:-	NBAT1;NONHSAG043159
l011825	chr6:25992640-26271353:+	NONHSAG043229;NONHSAG093064
l011838	chr6:26521728-26528653:+	NONHSAG043250
l011848	chr6:26956914-27059749:+	LINC00240;NONHSAG093078
l011873	chr6:28089898-28137670:-	NONHSAG043303
l011961	chr6:30723104-30724195:-	NONHSAG094075
l011964	chr6:30743789-30794324:+	HCG20;NONHSAG043433
l012032	chr6:32184732-32185882:-	NONHSAG043550;XXbac-BPG300A18.13
l012058	chr6:33142993-33148063:+	HCG24;NONHSAG043592
l012081	chr6:34235620-34236694:+	NONHSAG043625
l012103	chr6:35542393-35614756:+	NONHSAG043657
l012118	chr6:36673620-36675126:-	NONHSAG094129;PANDAR
l012179	chr6:41496104-41548621:-	NONHSAG043770
l012252	chr6:44299471-44300772:+	NONHSAG043851

Unified node ID	Genomic coordinates	Matched evidence ID[1]
l012282	chr6:47729826-47740741:-	RP11-550C4.6
l012302	chr6:52576785-52643081:+	NONHSAG043934;TRAM2-AS1
l012350	chr6:57907998-57961501:-	NONHSAG044022
l012369	chr6:63571004-63572408:-	NONHSAG044041
l012380	chr6:67878315-67889339:-	NONHSAG044064
l012402	chr6:71187160-71477490:-	LINC00472;NONHSAG094269
l012509	chr6:85659106-85679247:-	NONHSAG044283
l012557	chr6:89950115-89953187:+	NONHSAG044350
l012594	chr6:96548629-96694427:-	NONHSAG044406
l012607	chr6:98873724-98948006:-	NONHSAG044429
l012671	chr6:107957207-107974423:+	NONHSAG044533
l012720	chr6:111482712-111602930:+	NONHSAG093517
l012732	chr6:112116232-112166476:+	NONHSAG044624
l012753	chr6:113969700-114540720:+	NONHSAG044666
l012782	chr6:117608547-117675773:-	NONHSAG044709
l012819	chr6:123389420-123511509:+	NONHSAG044751;TRDN-AS1
l012845	chr6:127343626-127345327:+	NONHSAG044791
l013050	chr6:149796150-149836204:-	NONHSAG045131
l013066	chr6:151391543-151405304:+	NONHSAG045160
l013069	chr6:151656691-151807981:+	NONHSAG045163
l013071	chr6:151842602-152094551:+	NONHSAG045166
l013159	chr6:160003290-160007664:-	NONHSAG045306
l013209	chr6:165780269-165988048:-	NONHSAG045383
l013238	chr6:167781477-167827561:-	AFDN-DT;NONHSAG045430
l013301	chr7:176560-194641:-	NONHSAG046722

Unified node ID	Genomic coordinates	Matched evidence ID[1]
l013307	chr7:379359-382879:+	NONHSAG046730
l013333	chr7:1459761-1464707:+	NONHSAG046765
l013344	chr7:1727353-1742316:-	NONHSAG046781
l013352	chr7:2383697-2388652:+	AC004840.8
l013360	chr7:2944023-2947092:+	NONHSAG046811
l013376	chr7:4973984-5040675:+	NONHSAG046839
l013426	chr7:8261437-8400006:+	AC007009.1;NONHSAG096130
l013440	chr7:11180901-11520175:+	NONHSAG046953
l013586	chr7:27095646-27107474:+	HOTAIRM1;NONHSAG096227
l013587	chr7:27107776-27134302:+	NONHSAG047182
l013589	chr7:27129976-27155928:+	HOXA-AS3;NONHSAG096230
l013593	chr7:27184457-27194142:+	NONHSAG047191
l013596	chr7:27198574-27207260:+	NONHSAG047197
l013635	chr7:30510688-30595286:-	NONHSAG047260
l013670	chr7:34346511-34916824:-	NONHSAG047319
l013729	chr7:39947518-39950793:-	NONHSAG113761
l013741	chr7:41693883-41779388:+	INHBA-AS1;NONHSAG047433
l013760	chr7:43940894-44039008:-	NONHSAG047463
l013762	chr7:44039048-44044490:+	NONHSAG047469
l013763	chr7:44044639-44069456:+	NONHSAG047471
l013782	chr7:44977833-45000008:-	NONHSAG047507;SNHG15
l013811	chr7:47621938-47629893:+	NONHSAG047543
l013813	chr7:47761475-47770416:+	NONHSAG047544
l013815	chr7:47955364-47957314:-	NONHSAG047547
l013870	chr7:55019016-55211628:+	NONHSAG047619

Unified node ID	Genomic coordinates	Matched evidence ID[1]
l013871	chr7:55171672-55213723:-	EGFR-AS1;NONHSAG047620
l013927	chr7:63326066-63354457:-	NONHSAG047725
l013962	chr7:64794384-64858711:+	NONHSAG047792
l013967	chr7:64888512-64890894:-	NONHSAG047796
l013989	chr7:66119602-66165011:-	NONHSAG047830
l013991	chr7:66375601-66493606:-	LINC00174;NONHSAG047840
l013995	chr7:66493686-66504420:+	NONHSAG047842
l013999	chr7:66653364-66775307:+	NONHSAG047851;RP4-756H11.3
l014065	chr7:73735037-73736054:+	LINC00035
l014070	chr7:74028157-74070180:+	NONHSAG047951
l014072	chr7:74058904-74062308:-	ELN-AS1
l014086	chr7:74881789-74890612:-	NONHSAG047973
l014087	chr7:74890774-74921138:+	NONHSAG047974
l014137	chr7:77477983-77697068:-	NONHSAG048057
l014164	chr7:81690355-82030612:+	NONHSAG048101
l014174	chr7:84926636-84940256:-	NONHSAG048114
l014220	chr7:91939876-91941137:-	NONHSAG048189
l014221	chr7:91940839-92110673:+	NONHSAG048191
l014228	chr7:92457563-92519877:+	NONHSAG048201
l014246	chr7:94071758-94396576:-	NONHSAG048228
l014249	chr7:94417723-94430395:+	NONHSAG048231
l014273	chr7:96950684-97014088:-	DLX6-AS1;NONHSAG048270
l014299	chr7:99013164-99048240:+	NONHSAG048302
l014313	chr7:99558667-99602800:+	NONHSAG048323
l014340	chr7:100336078-100367831:+	NONHSAG048354

Unified node ID	Genomic coordinates	Matched evidence ID[1]
l014389	chr7:102483343-102543764:-	RP11-514P8.8
l014396	chr7:102973353-102988857:+	NFE4;NONHSAG048439
l014414	chr7:104894544-105014321:-	NONHSAG048464
l014508	chr7:115789728-115791329:+	NONHSAG048598
l014510	chr7:116194587-116240781:-	NONHSAG048602
l014511	chr7:116235755-116499547:-	NONHSAG048603
l014515	chr7:116563593-116614820:-	NONHSAG048610
l014525	chr7:117447909-117504308:+	NONHSAG048623
l014526	chr7:117560732-117564676:-	NONHSAG048624
l014527	chr7:117604790-117647416:-	NONHSAG048626
l014538	chr7:120166442-120187051:+	NONHSAG048641
l014549	chr7:122144404-122314516:+	FEZF1-AS1;NONHSAG096627
l014561	chr7:123748541-123771827:+	NONHSAG048682
l014571	chr7:124925769-125179315:+	NONHSAG048695
l014629	chr7:129548103-129611675:-	NONHSAG048781
l014653	chr7:130791263-131190429:-	NONHSAG097344
l014654	chr7:130853719-131109637:+	NONHSAG048817
l014656	chr7:131309296-131328253:-	NONHSAG097347
l014789	chr7:143379631-143381218:-	NONHSAG049037
l014799	chr7:144194857-144312406:+	NONHSAG049050
l014800	chr7:144238572-144356181:-	NONHSAG049051
l014821	chr7:148987526-148989432:+	GHET1
l014848	chr7:150031633-150079072:+	NONHSAG049119
l014857	chr7:150362626-150373296:-	NONHSAG049129
l014873	chr7:151074741-151076530:-	NONHSAG049157

Unified node ID	Genomic coordinates	Matched evidence ID[1]
l014894	chr7:151876292-151879834:+	NONHSAG049185
l014898	chr7:152113137-152122470:-	NONHSAG049191
l014974	chr7:157007749-157053772:+	MNX1-AS1;NONHSAG049288
l014983	chr7:157281535-157282686:-	NONHSAG049303
l015025	chr8:737627-1357934:+	NONHSAG049356
l015066	chr8:6615602-6708216:-	NONHSAG049421
l015115	chr8:9886104-9907034:-	NONHSAG049507
l015201	chr8:17131180-17149790:+	NONHSAG049626
l015220	chr8:18059643-18084944:-	NONHSAG049655
l015221	chr8:18084227-18127457:+	NONHSAG098653
l015286	chr8:23224470-23231085:+	NONHSAG049754
l015323	chr8:26291507-26372680:+	NONHSAG049801
l015387	chr8:30375389-30385402:-	NONHSAG049901
l015508	chr8:41509592-41578421:-	NONHSAG050073
l015527	chr8:42416474-42546758:-	NONHSAG050104
l015562	chr8:47941425-47943670:+	NONHSAG050167
l015563	chr8:47942580-47944778:+	NONHSAG050168
l015626	chr8:56067294-56074509:+	NONHSAG050272
l015627	chr8:56073057-56074492:-	NONHSAG050273
l015634	chr8:56494281-56559824:-	NONHSAG050284
l015680	chr8:61684034-61714575:-	NONHSAG050345
l015734	chr8:66921683-66926398:-	NONHSAG050423
l015749	chr8:68911802-69104201:-	NONHSAG050445
l015774	chr8:71828166-72118393:+	NONHSAG050493
l015790	chr8:73420003-73441527:+	NONHSAG050518

Unified node ID	Genomic coordinates	Matched evidence ID[1]
l015899	chr8:86098964-86154225:-	NONHSAG050670
l015935	chr8:91048658-91070190:-	NONHSAG050725
l015960	chr8:93754843-93819234:+	NONHSAG050769
l015971	chr8:94553460-94594794:+	NONHSAG050786
l015988	chr8:95958700-95993224:-	NONHSAG050815
l016058	chr8:101460004-101493381:-	NONHSAG050929
l016083	chr8:103398637-103415335:-	NONHSAG050985
l016099	chr8:105533416-105543204:-	NONHSAG051010
l016100	chr8:105546088-106060524:-	NONHSAG051015;ZFPM2-AS1
l016193	chr8:120812218-120813359:+	NONHSAG099082
l016216	chr8:123193506-123202744:-	NONHSAG051166
l016238	chr8:124452955-124474992:-	NONHSAG051196
l016268	chr8:126552461-127419050:+	CCAT2;NONHSAG051250;NONHSAG099120
l016270	chr8:126766160-126847016:-	NONHSAG051245
l016271	chr8:127072693-127482140:-	NONHSAG099742
l016273	chr8:127629674-127735897:-	NONHSAG051255
l016275	chr8:127794512-128196509:+	NONHSAG051260;NONHSAG051263;NONHSAG099126;PVT1
l016276	chr8:127811245-128101256:-	NONHSAG051259
l016279	chr8:128405269-128564679:-	NONHSAG051266
l016295	chr8:130052103-130443848:-	NONHSAG051291
l016336	chr8:134715819-134832309:-	NONHSAG099774
l016406	chr8:142657459-142682723:-	JRK;NONHSAG051448
l016412	chr8:142763046-142766428:+	NONHSAG051454
l016430	chr8:143279655-143282651:-	NONHSAG051484
l016433	chr8:143366108-143368792:-	NONHSAG114445;RHPN1-AS1

Unified node ID	Genomic coordinates	Matched evidence ID[1]
l016452	chr8:143716268-143719174:-	NONHSAG051514
l016454	chr8:143730164-143790645:+	RP11-429J17.8
l016455	chr8:143758152-143771870:-	NONHSAG051517
l016462	chr8:143922963-143924660:+	NONHSAG051523
l016476	chr8:144314583-144326910:-	NONHSAG051542
l016496	chr8:144511287-144517845:-	NONHSAG051567
l016497	chr8:144512566-144513672:+	NONHSAG051568
l016526	chr9:100595-115772:-	NONHSAG051603
l016614	chr9:8857811-8865021:+	NONHSAG051770
l016692	chr9:21364604-21591906:-	MIR31HG;NONHSAG102572
l016700	chr9:21967751-21995301:-	NONHSAG051898
l016701	chr9:21994129-22876946:+	CDKN2B-AS1;NONHSAG101229
l016732	chr9:27235683-27282793:-	NONHSAG051958
l016788	chr9:33722372-33818836:-	NONHSAG052046
l016811	chr9:34661902-34666174:-	NONHSAG052082
l016823	chr9:35073834-35080004:-	NONHSAG052097
l016839	chr9:35657750-35658018:-	NONHSAG052119
l016860	chr9:36840558-37026575:-	NONHSAG052149
l016862	chr9:37002634-37008040:+	NONHSAG052151
l016879	chr9:37759233-37832117:-	NONHSAG052179
l016923	chr9:40992248-41217197:+	NONHSAG052454
l016924	chr9:41009306-41035565:-	NONHSAG052459
l017008	chr9:68305988-68330626:+	NONHSAG052491;NONHSAG052492
l017011	chr9:68374875-68406500:-	NONHSAG052495
l017022	chr9:69296670-69311111:-	BANCR;NONHSAG052510

Unified node ID	Genomic coordinates	Matched evidence ID[1]
l017023	chr9:69394305-69398837:-	NONHSAG052511
l017085	chr9:76764435-76787570:+	NONHSAG052609
l017120	chr9:83219183-83287463:+	NONHSAG052680;RP11-439K3.1
l017125	chr9:83817568-83868532:+	NONHSAG052686
l017141	chr9:85756041-85805907:-	NONHSAG052709
l017151	chr9:86948043-87003459:+	NONHSAG052726
l017215	chr9:92670301-92674756:+	NONHSAG052853
l017252	chr9:94554598-94586674:+	NONHSAG052908
l017256	chr9:95099053-95426881:-	NONHSAG052925
l017260	chr9:95442979-95517057:-	NONHSAG052931;NONHSAG052933
l017262	chr9:95463608-95470019:+	NONHSAG052929
l017264	chr9:95506187-95569883:+	NONHSAG102979
l017268	chr9:95758609-95875979:-	NONHSAG052943
l017312	chr9:97986550-97987657:-	NONHSAG053007
l017318	chr9:98625595-98877362:-	NONHSAG053014
l017328	chr9:99388682-99399668:-	NONHSAG053028
l017329	chr9:99396986-99906614:-	NONHSAG053033
l017358	chr9:103184971-103206562:+	NONHSAG053082
l017377	chr9:105525205-105558465:-	NONHSAG053108
l017406	chr9:108868635-108878123:-	NONHSAG053162
l017457	chr9:113103337-113111677:-	NONHSAG053228
l017578	chr9:124770122-124774967:+	NONHSAG053419
l017600	chr9:126407773-126410504:-	NONHSAG053456
l017624	chr9:127811129-127854773:-	NONHSAG053499;NONHSAG053502
l017625	chr9:127815943-127822867:+	NONHSAG053500

Unified node ID	Genomic coordinates	Matched evidence ID[1]
l017654	chr9:128411750-128437351:+	NONHSAG101725
l017658	chr9:128523271-128553507:+	NONHSAG053546
l017659	chr9:128528539-128552494:-	NONHSAG053547
l017692	chr9:129640450-129641527:+	NONHSAG053590
l017700	chr9:129914190-129936541:+	NONHSAG053602
l017720	chr9:130933803-130945527:+	NONHSAG053626
l017724	chr9:131132851-131167505:-	NONHSAG053631
l017737	chr9:131516557-131522230:-	NONHSAG053651
l017782	chr9:134025468-134032854:+	NONHSAG053716
l017784	chr9:134054269-134059051:+	NONHSAG053719
l017799	chr9:134819043-134872636:-	NONHSAG053748
l017836	chr9:136542880-136543836:+	NONHSAG053796
l017839	chr9:136633289-136663334:-	NONHSAG053802
l017871	chr9:137054132-137064581:+	NONHSAG053842
l017989	chr10:4993332-4997381:+	NONHSAG005084
l018030	chr10:6682512-6882907:+	NONHSAG005153
l018044	chr10:8015564-8057043:-	GATA3-AS1;NONHSAG061096
l018149	chr10:18513105-18545651:-	NONHSAG005339
l018171	chr10:21534299-21673691:+	NONHSAG005377
l018191	chr10:23343942-23362217:+	NONHSAG005403
l018239	chr10:28507053-28533601:-	NONHSAG005482;WAC-AS1
l018299	chr10:33055798-33116817:-	IATPR
l018363	chr10:41843734-41856931:+	NONHSAG005669
l018380	chr10:43077259-43100655:+	NONHSAG005699
l018388	chr10:43407849-43423372:+	NONHSAG005711

Unified node ID	Genomic coordinates	Matched evidence ID[1]
l018395	chr10:43628816-43674704:+	NONHSAG005721;NONHSAG005728
l018399	chr10:43777560-43778869:-	NONHSAG005732
l018405	chr10:44282488-44295210:-	NONHSAG005742
l018435	chr10:45853469-45892483:-	NONHSAG005780
l018473	chr10:47908063-47997742:-	NONHSAG005838
l018494	chr10:49419234-49472933:+	NONHSAG005866
l018519	chr10:52167103-52425415:-	NONHSAG005920;PRKG1-AS1
l018574	chr10:63168064-63191060:-	NONHSAG006007
l018598	chr10:66079242-66121129:+	NONHSAG006049
l018599	chr10:66894109-67012153:-	NONHSAG006050
l018602	chr10:67539625-67665624:-	NONHSAG006054
l018607	chr10:68233222-68242380:-	LINC02640;NONHSAG006071
l018634	chr10:69215332-69232490:-	RP11-227H15.4
l018672	chr10:71816298-71819543:-	NONHSAG006163
l018702	chr10:73495525-73520070:+	NONHSAG006204
l018707	chr10:73647528-73676496:+	NONHSAG006212
l018722	chr10:74096165-74102547:-	NONHSAG006238
l018742	chr10:75398580-75412482:+	NONHSAG060586;ZNF503-AS2
l018762	chr10:77925668-77930242:+	NONHSAG006301
l018777	chr10:79662739-79826770:-	NONHSAG006341
l018788	chr10:80207709-80229592:+	NONHSAG006362
l018793	chr10:80519576-80543244:-	NONHSAG061404;RP11-137H2.4
l018850	chr10:87862637-87971930:+	NONHSAG006457
l018851	chr10:87863570-87864698:-	NONHSAG006454
l018860	chr10:88879733-88975153:+	NONHSAG006474

Unified node ID	Genomic coordinates	Matched evidence ID[1]
l018863	chr10:88991420-88992975:-	NONHSAG006477
l018897	chr10:91781412-91798336:-	NONHSAG006526
l018967	chr10:97356356-97426076:-	NONHSAG006633
l019001	chr10:100361639-100408142:+	NONHSAG006678
l019012	chr10:100969457-100987515:-	NONHSAG006692
l019018	chr10:101044874-101061649:-	NONHSAG061492
l019036	chr10:101818767-101829599:+	NONHSAG006735
l019047	chr10:102438135-102461106:+	NONHSAG006756
l019054	chr10:102830755-102834517:+	NONHSAG006763
l019062	chr10:103193300-103196246:+	NONHSAG006772
l019120	chr10:110868742-110881380:-	NONHSAG006865;PDCD4-AS1
l019182	chr10:116669953-116681929:+	NONHSAG006945
l019200	chr10:118045861-118216096:+	NONHSAG006966
l019281	chr10:124447151-124450048:+	NONHSAG007109
l019400	chr11:287189-288996:+	NONHSAG007295
l019418	chr11:563689-568542:-	MIR210HG;NONHSAG007324
l019456	chr11:1664168-1667856:+	NONHSAG007384
l019489	chr11:2444653-2849105:+	NONHSAG007430
l019490	chr11:2606133-2699998:-	NONHSAG007431
l019491	chr11:2840134-2862191:-	NONHSAG007434
l019502	chr11:3057535-3064707:+	AC108448.3
l019507	chr11:3336720-3340630:+	NONHSAG007455
l019513	chr11:3424393-3605899:-	NONHSAG007463
l019524	chr11:4175884-4204965:+	LINC02749;NONHSAG007487
l019564	chr11:6608666-6610135:-	NONHSAG007538

Unified node ID	Genomic coordinates	Matched evidence ID[1]
l019608	chr11:8964202-8979199:+	NONHSAG007602
l019624	chr11:9758291-9811319:+	NONHSAG007631
l019664	chr11:12822434-12823667:-	NONHSAG007692
l019674	chr11:13668658-13732346:+	FAR1-IT1;NONHSAG007708
l019680	chr11:14262845-14273692:-	NONHSAG007715;SPON1-AS1
l019776	chr11:23761261-23808259:-	NONHSAG007868
l019828	chr11:31305684-31322295:+	NONHSAG007961
l019841	chr11:32389057-32396403:-	NONHSAG007983
l019842	chr11:32434074-32458769:+	NONHSAG007984
l019862	chr11:33774562-33811369:+	FBXO3-DT;NONHSAG008015
l019876	chr11:34570875-34573982:+	LINC02707;NONHSAG008037
l019889	chr11:35418961-35483459:+	NONHSAG008056
l019959	chr11:45307015-45462682:+	NONHSAG008167
l019981	chr11:46237388-46245049:+	NONHSAG008199
l019993	chr11:46846410-46874416:+	NONHSAG008220
l020062	chr11:57476492-57477534:-	NONHSAG008354
l020165	chr11:62049862-62123841:-	LINC02733
l020175	chr11:62545561-62547699:+	NONHSAG008517
l020194	chr11:62771119-62792006:-	NONHSAG008540
l020202	chr11:62851873-62856011:-	NONHSAG008552
l020223	chr11:64166651-64185716:-	NONHSAG008589
l020244	chr11:64500845-64505387:+	AP005273.1;NONHSAG008615
l020265	chr11:65110713-65111695:-	NONHSAG008654
l020279	chr11:65416581-65450093:+	NONHSAG008670
l020282	chr11:65496266-65509085:+	NONHSAG008675

Unified node ID	Genomic coordinates	Matched evidence ID[1]
l020293	chr11:65655707-65659743:-	NONHSAG008693
l020323	chr11:66267634-66268129:-	NONHSAG008726
l020324	chr11:66269831-66278525:-	NONHSAG008728
l020348	chr11:66677805-66685321:+	NONHSAG008759
l020413	chr11:68612898-68616711:-	NONHSAG008859
l020463	chr11:71448540-71453016:+	NONHSAG008937
l020471	chr11:71791572-71819787:-	NONHSAG008952
l020481	chr11:72014290-72038409:+	NONHSAG008966
l020512	chr11:73395558-73396436:-	NONHSAG009021
l020527	chr11:73963656-73970288:-	NONHSAG009039
l020536	chr11:74397548-74485743:+	NONHSAG009059;NONHSAG009062
l020578	chr11:76210955-76216025:-	NONHSAG009124
l020583	chr11:76427228-76445764:-	NONHSAG009130
l020585	chr11:76607363-76630465:-	LINC02757;NONHSAG009133
l020586	chr11:76654168-76656713:-	NONHSAG009134
l020590	chr11:76712395-76719608:-	RP11-672A2.1
l020614	chr11:78022560-78023691:+	NONHSAG009174
l020617	chr11:78139653-78187685:+	KCTD21-AS1;NONHSAG009180
l020627	chr11:79191557-79193160:+	NONHSAG009193
l020655	chr11:83184002-83194248:-	NONHSAG009228;PCF11-AS1
l020862	chr11:108222803-108369102:+	NONHSAG009544;NONHSAG009546
l020901	chr11:112037323-112063283:+	NONHSAG009613
l020917	chr11:112959274-112963488:-	NONHSAG009636
l020972	chr11:117289497-117293571:+	NONHSAG009728
l020987	chr11:118133376-118145469:-	NONHSAG009747

Unified node ID	Genomic coordinates	Matched evidence ID[1]
l020991	chr11:118339073-118342744:-	NONHSAG009755
l020993	chr11:118344385-118353332:-	NONHSAG009757
l021009	chr11:118791024-118797646:+	NONHSAG009782
l021076	chr11:122028203-122556721:-	NONHSAG063700
l021097	chr11:123653877-123675569:+	NONHSAG009913
l021112	chr11:124758840-124772741:+	NONHSAG009938
l021117	chr11:124883690-124887790:+	NONHSAG009945
l021123	chr11:125094369-125111763:-	NONHSAG009950
l021139	chr11:125824516-125887572:-	NONHSAG009968
l021156	chr11:126289020-126294255:-	NONHSAG009989
l021182	chr11:128691671-128696024:-	NONHSAG010021
l021242	chr11:135061780-135075908:-	NONHSAG010117
l021282	chr12:1917847-1922868:+	NONHSAG010179
l021284	chr12:1970771-2697950:+	NONHSAG010186;NONHSAG010189
l021285	chr12:2220281-2223481:-	NONHSAG010184
l021289	chr12:2603349-2607440:-	CACNA1C-AS3;NONHSAG010190
l021290	chr12:2668499-2672221:-	NONHSAG010193
l021291	chr12:2676000-2691201:-	NONHSAG010194
l021292	chr12:2695764-2812913:-	NONHSAG010200
l021303	chr12:3291438-3366104:-	NONHSAG065252;RP5-1063M23.1
l021384	chr12:6914449-6923696:+	NONHSAG010348
l021389	chr12:6962023-6965382:+	MIR200CHG;NONHSAG010352
l021422	chr12:8295736-8396803:-	NONHSAG010405
l021429	chr12:8654424-8669012:+	NONHSAG010416
l021523	chr12:12890781-13040680:+	GPRC5D-AS1;NONHSAG010543

Unified node ID	Genomic coordinates	Matched evidence ID[1]
l021603	chr12:21662312-21773899:+	NONHSAG010657
l021604	chr12:21827209-21887958:+	NONHSAG010658
l021637	chr12:25924171-25959766:-	NONHSAG010716
l021677	chr12:28062585-28190912:-	NONHSAG065364
l021697	chr12:30755040-30881888:+	NONHSAG010792
l021700	chr12:31019433-31073848:-	DDX11-AS1;NONHSAG010798
l021783	chr12:42962470-42967353:+	NONHSAG010940
l021813	chr12:46371189-46374684:+	NONHSAG010986
l021815	chr12:46383040-46883354:+	NONHSAG010989
l021838	chr12:47882648-47901525:+	NONHSAG011028
l021860	chr12:48766193-48789089:-	NONHSAG011057
l021869	chr12:48995149-48996334:+	NONHSAG011070
l021879	chr12:49110473-49188484:+	NONHSAG011084;NONHSAG011086
l021883	chr12:49265155-49273346:-	NONHSAG011090
l021903	chr12:49949962-49975539:-	NONHSAG011121
l021906	chr12:50112196-50165618:+	NONHSAG011125
l021960	chr12:53012883-53054801:-	NONHSAG011211
l021970	chr12:53298651-53300583:-	NONHSAG011230;RP11-680A11.5
l021991	chr12:53962307-53974957:-	NONHSAG011264
l022023	chr12:55729077-55738937:+	NONHSAG066253
l022031	chr12:55954104-55973317:-	NONHSAG011337
l022036	chr12:56010090-56041806:-	NONHSAG011343
l022054	chr12:56300038-56317992:+	NONHSAG011375
l022098	chr12:57612117-57621903:-	NONHSAG011439
l022101	chr12:57693840-57728356:+	NONHSAG064784

Unified node ID	Genomic coordinates	Matched evidence ID[1]
l022102	chr12:57694131-57721510:-	NONHSAG011446
l022155	chr12:63804738-63822219:-	NONHSAG011518;RXYLT1-AS1
l022161	chr12:64038561-64097619:-	NONHSAG011524
l022204	chr12:66793058-67096410:-	NONHSAG011576;RP11-123O10.4
l022232	chr12:69212107-69224242:+	NONHSAG011633
l022243	chr12:69901917-70244533:-	NONHSAG011658
l022330	chr12:79934900-79950423:+	NONHSAG011790;PPP1R12A-AS1
l022369	chr12:88055197-88059058:+	NONHSAG011854
l022380	chr12:89524030-89603134:+	NONHSAG011870;POC1B-AS1
l022421	chr12:93677374-93894840:+	NONHSAG011935
l022467	chr12:96985190-97598415:+	NONHSAG064962;RMST
l022478	chr12:98510417-98516454:-	NONHSAG012025
l022507	chr12:101594970-101692681:+	NONHSAG012076
l022508	chr12:101646719-101664492:-	NONHSAG012077
l022519	chr12:102197584-102463502:+	NONHSAG012099
l022657	chr12:110942939-110957813:-	NONHSAG012297
l022662	chr12:111443484-111599676:-	NONHSAG012303
l022687	chr12:112418350-112509918:+	NONHSAG012339
l022706	chr12:113679458-113773683:-	NONHSAG012367
l022713	chr12:114077102-114195435:+	NONHSAG012375;RP11-100F15.1
l022742	chr12:116517857-116554381:+	LINC00173;NONHSAG012423
l022781	chr12:119173990-119177553:-	NONHSAG012476
l022797	chr12:120194916-120215485:+	NONHSAG012505
l022821	chr12:120888868-120980968:-	HNF1A-AS1;NONHSAG012534
l022826	chr12:121195247-121788902:-	NONHSAG012540

Unified node ID	Genomic coordinates	Matched evidence ID[1]
l022830	chr12:121580332-121597226:+	KDM2B-DT;NONHSAG012549
l022834	chr12:121795258-121803906:-	NONHSAG012555
l022862	chr12:122974579-122982914:+	NONHSAG012594;RP11-197N18.2
l022878	chr12:123570392-123602035:-	NONHSAG012614;TMED2-DT
l022880	chr12:123707142-123712218:-	NONHSAG012617
l022973	chr12:130412628-130430661:+	NONHSAG012751
l023010	chr12:131910651-131929646:-	NONHSAG012791
l023157	chr13:23888748-23897388:+	NONHSAG013005
l023190	chr13:26337566-26400515:+	NONHSAG013060
l023215	chr13:28014485-28023421:-	NONHSAG013095
l023216	chr13:28132304-28139093:-	NONHSAG013098
l023225	chr13:28701397-28718957:-	NONHSAG013109
l023410	chr13:44397724-44410786:-	NONHSAG013388
l023440	chr13:46052492-46122699:+	NONHSAG013431
l023464	chr13:48286977-48303723:-	NONHSAG013475;RB1-DT
l023490	chr13:49912701-50125541:-	NONHSAG013515
l023499	chr13:50081724-50906856:+	NONHSAG013534
l023523	chr13:52132642-52159861:-	NONHSAG013568
l023616	chr13:67326312-67379977:+	NONHSAG013708
l023626	chr13:70107177-70139753:+	NONHSAG013730
l023663	chr13:75549772-75859870:+	NONHSAG013790
l023678	chr13:77042473-77327094:-	NONHSAG013809
l023719	chr13:84562363-84563236:+	LINC00333
l023739	chr13:87801035-87811232:-	NONHSAG013912
l023742	chr13:88142866-88236082:+	LINC00373;NONHSAG013917

Unified node ID	Genomic coordinates	Matched evidence ID[1]
l023757	chr13:90493287-90535595:+	NONHSAG013940
l023759	chr13:90890953-90926597:-	NONHSAG013942
l023760	chr13:91087002-91099519:-	LINC00380;NONHSAG013944
l023762	chr13:91119705-91211698:-	NONHSAG013946
l023764	chr13:91347686-91397097:+	NONHSAG068402
l023765	chr13:91398620-92873682:+	GPC5-IT1;NONHSAG013954
l023794	chr13:95648710-95677367:-	DNAJC3-DT;NONHSAG013992
l023864	chr13:102394577-102395705:+	NONHSAG014098
l023872	chr13:102851198-102856068:+	NONHSAG014112
l023889	chr13:106374476-106384411:+	LINC00460;NONHSAG014130
l023978	chr13:113297238-113361869:+	NONHSAG014267
l023991	chr13:113815609-113845746:+	NONHSAG014289
l024018	chr14:19100005-19353339:-	NONHSAG014332
l024032	chr14:20342975-20343685:-	NONHSAG014362
l024041	chr14:20474788-20477089:-	NONHSAG014372
l024042	chr14:20476488-20477087:+	NONHSAG014373
l024046	chr14:20685345-20799949:-	NONHSAG014381
l024109	chr14:23386029-23418763:+	NONHSAG068639
l024110	chr14:23412737-23435638:-	NONHSAG014496
l024112	chr14:23511759-23571796:+	NONHSAG014502
l024140	chr14:24241033-24242427:+	NONHSAG014546
l024149	chr14:24595722-24658171:+	NONHSAG014562;RP11-104E19.1
l024170	chr14:28772703-28813453:+	NONHSAG014591
l024223	chr14:34545807-34562387:+	NONHSAG014672
l024227	chr14:34754020-34983127:-	NONHSAG069147

Unified node ID	Genomic coordinates	Matched evidence ID[1]
l024228	chr14:34874230-34879227:+	NONHSAG014679
l024260	chr14:36473206-36519521:-	NONHSAG014730
l024353	chr14:49853639-49862853:+	NONHSAG014890
l024354	chr14:49860313-49868199:-	NONHSAG014891
l024365	chr14:50396309-50419425:+	NONHSAG014914
l024454	chr14:55487404-55581068:-	KTN1-AS1;NONHSAG015044
l024527	chr14:60639215-60727514:+	NONHSAG015163
l024562	chr14:64422934-64448557:-	NONHSAG015215
l024574	chr14:65082033-65101628:+	NONHSAG015234
l024619	chr14:68622794-68685565:-	NONHSAG069277
l024660	chr14:70593313-70641785:-	CTD-2540L5.5;NONHSAG015352
l024700	chr14:73494151-73530610:+	NONHSAG015417
l024724	chr14:74285268-74303055:-	NONHSAG015454
l024729	chr14:74494045-74495428:+	NONHSAG015461
l024756	chr14:75561587-75579657:-	NONHSAG069876
l024760	chr14:75902135-76084585:+	NONHSAG015505
l024825	chr14:81012098-81170439:-	NONHSAG015591
l024851	chr14:85197622-85239474:-	NONHSAG015623
l024859	chr14:86006892-86062981:-	LINC02316
l024898	chr14:90402522-90405235:-	NONHSAG015692
l024917	chr14:91417940-91437572:+	NONHSAG015723
l024965	chr14:95086227-95158010:-	NONHSAG015787
l024966	chr14:95157438-95179990:+	NONHSAG015788
l025057	chr14:100779205-101038859:+	MEG3;NONHSAG069030
l025082	chr14:101791146-101810321:-	NONHSAG015961

Unified node ID	Genomic coordinates	Matched evidence ID[1]
l025123	chr14:103679828-103684941:-	NONHSAG016021
l025152	chr14:104769348-104795759:-	NONHSAG016068
l025181	chr14:105527918-106874878:-	AL928742.12
l025183	chr14:105582801-106592970:+	NONHSAG016118
l025189	chr15:20344735-20359175:+	NONHSAG016199
l025233	chr15:24978406-25420336:+	NONHSAG016302;NONHSAG016307;SNHG14
l025286	chr15:30004943-30045899:-	NONHSAG016395
l025291	chr15:30196035-30214540:+	NONHSAG016403
l025345	chr15:34255285-34257832:+	NONHSAG016486
l025346	chr15:34336407-34340368:+	NONHSAG016487
l025406	chr15:40038278-40076539:+	NONHSAG070257;SRP14-AS1
l025409	chr15:40088831-40089386:+	NONHSAG016576
l025431	chr15:40729505-40731359:+	NONHSAG016611
l025437	chr15:40834723-40844636:-	NONHSAG016618;SPINT1-AS1
l025439	chr15:40906297-40910337:+	NONHSAG016623
l025517	chr15:44402684-44427144:-	NONHSAG016733
l025521	chr15:44524058-44537461:-	EIF3J-DT;NONHSAG016741
l025525	chr15:44711486-44718158:+	NONHSAG016749
l025539	chr15:45200324-45200632:-	NONHSAG016771
l025570	chr15:48289905-48331856:-	NONHSAG016822
l025617	chr15:51377284-51385397:+	NONHSAG016893
l025699	chr15:58434889-58498735:-	NONHSAG017020
l025721	chr15:59688509-59696418:+	NONHSAG017060
l025845	chr15:66928484-66957324:-	LINC02206;NONHSAG017232
l025849	chr15:67063762-67195173:+	NONHSAG017238

Unified node ID	Genomic coordinates	Matched evidence ID[1]
l025859	chr15:67834307-67838880:-	NONHSAG017254
l025877	chr15:69390360-69462856:-	NONHSAG017282
l025880	chr15:69462964-69575770:+	NONHSAG017288
l025922	chr15:72278866-72351794:+	NONHSAG017344
l025924	chr15:72376112-72378788:+	NONHSAG017347
l025926	chr15:72406415-72475168:-	NONHSAG017349;TMEM202-AS1
l025942	chr15:73335139-73342387:+	NONHSAG017376
l025943	chr15:73368271-73368914:-	NONHSAG017377
l025944	chr15:73560013-73634134:-	NONHSAG017379
l025972	chr15:74461264-74481306:+	NONHSAG017417
l026012	chr15:75737723-75763376:+	NONHSAG017486
l026021	chr15:76339608-76342063:-	NONHSAG017504
l026063	chr15:78589122-78591276:-	NONHSAG017567
l026123	chr15:82647766-82692820:+	NONHSAG017666
l026186	chr15:85743855-85758725:-	NONHSAG017767
l026197	chr15:87576920-87579866:+	NONHSAG017783
l026257	chr15:90620006-90717141:-	NONHSAG017882
l026261	chr15:90790037-90790661:-	NONHSAG017890
l026266	chr15:90881281-90883459:-	NONHSAG017899
l026349	chr15:98572364-98648337:-	IRAIN
l026350	chr15:98645661-98651222:-	NONHSAG018053
l026353	chr15:98880658-98893535:-	NONHSAG018054
l026355	chr15:99014677-99031053:+	NONHSAG018058
l026388	chr15:101042266-101086495:-	NONHSAG018110
l026435	chr16:547184-553847:-	NONHSAG018180

Unified node ID	Genomic coordinates	Matched evidence ID[1]
l026453	chr16:689000-692554:+	NONHSAG018203
l026481	chr16:1260951-1263845:-	NONHSAG018236
l026532	chr16:2112274-2113561:+	NONHSAG018313
l026541	chr16:2240486-2241818:+	NONHSAG018322
l026542	chr16:2267880-2281329:+	NONHSAG018326
l026555	chr16:2537978-2603190:+	NONHSAG018341
l026557	chr16:2571569-2572353:-	NONHSAG018344
l026580	chr16:2939418-2956146:-	LA16c-321D4.2;NONHSAG018372
l026581	chr16:2972792-2980473:-	LA16c-321D4.2
l026584	chr16:2987314-3003501:+	LA16c-380H5.3;LINC00514;NONHSAG071444
l026612	chr16:3551780-3588670:+	NONHSAG018414;NONHSAG018415
l026613	chr16:3601120-3609554:+	NONHSAG018416
l026614	chr16:3611747-3643095:+	NONHSAG018417
l026617	chr16:3660741-3665472:+	NONHSAG018420
l026642	chr16:4696511-4734162:-	NONHSAG018461
l026679	chr16:8962147-8967363:+	NONHSAG018530
l026719	chr16:10864913-10888752:-	NONHSAG018586
l026739	chr16:11818292-11828845:-	NONHSAG018621
l026748	chr16:12093626-12095307:-	NONHSAG018635
l026760	chr16:13792902-13808052:-	NONHSAG018656
l026763	chr16:13928903-13935635:-	NONHSAG018659
l026792	chr16:15643266-15734691:+	NONHSAG018709
l026793	chr16:15703134-15858438:-	NONHSAG018707
l026794	chr16:15741150-15741791:+	NONHSAG018711
l026864	chr16:21120757-21130108:+	NONHSAG018829

Unified node ID	Genomic coordinates	Matched evidence ID[1]
l026908	chr16:23407869-23409512:+	NONHSAG018889
l026910	chr16:23452718-23469001:+	NONHSAG018893
l026915	chr16:23605840-23624107:+	NONHSAG018903
l026970	chr16:28282251-28293141:-	NONHSAG018980
l026985	chr16:28868383-28879921:-	NONHSAG019003
l026993	chr16:28973961-28978824:-	NONHSAG019012
l026994	chr16:28974803-28990784:+	RP11-264B17.3
l026995	chr16:28989139-28990778:-	NONHSAG019014
l026998	chr16:29074795-29403423:+	NONHSAG019021;RRN3P2;SNX29P2
l027000	chr16:29208005-29221040:-	NONHSAG019023
l027007	chr16:29455104-29464966:+	NONHSAG019031;SLX1B-SULT1A4
l027016	chr16:29806495-29807732:-	NONHSAG019049
l027017	chr16:29808635-29821252:-	NONHSAG019051
l027037	chr16:30183504-30184957:-	NONHSAG019079
l027044	chr16:30354664-30363713:+	CD2BP2-DT;NONHSAG019090
l027062	chr16:30696335-30699060:-	NONHSAG019115
l027064	chr16:30740666-30751381:+	RP11-2C24.4
l027074	chr16:30948385-30956511:+	AC135048.13
l027085	chr16:31118077-31118747:+	NONHSAG019134
l027105	chr16:31487369-31488492:-	NONHSAG019158
l027141	chr16:32882585-32888874:+	NONHSAG019221
l027161	chr16:34153597-34175493:+	NONHSAG019270
l027189	chr16:46622860-46624451:-	NONHSAG019332
l027212	chr16:48164951-48167238:-	NONHSAG019370
l027259	chr16:51054727-51056399:-	NONHSAG019439

Unified node ID	Genomic coordinates	Matched evidence ID[1]
l027282	chr16:53035689-53052897:-	RP11-467J12.4
l027289	chr16:53701691-54158512:+	NONHSAG019493
l027298	chr16:54844648-54931354:-	CRNDE;NONHSAG072176
l027344	chr16:57450784-57452836:+	NONHSAG019569
l027378	chr16:58733862-59199395:+	NONHSAG019613
l027407	chr16:65232003-65455451:+	NONHSAG019667
l027410	chr16:65601100-65601698:-	NONHSAG019670
l027444	chr16:67028983-67101058:+	NONHSAG019711
l027479	chr16:67842682-67843149:+	NONHSAG019767
l027507	chr16:68737291-68835542:+	NONHSAG019814
l027508	chr16:68812585-68823526:-	NONHSAG019813
l027529	chr16:69976296-70065948:-	NONHSAG019848
l027531	chr16:70137654-70173505:-	NONHSAG019852
l027552	chr16:70986849-71093667:+	NONHSAG019878
l027570	chr16:71723179-71724426:+	NONHSAG019898
l027604	chr16:74305108-74335346:-	NONHSAG019967
l027661	chr16:78193343-78241989:-	NONHSAG020053;WWOX-AS1
l027669	chr16:79207717-79229497:-	NONHSAG020066
l027671	chr16:79599610-79620099:+	NONHSAG020068
l027677	chr16:80154978-80572808:-	NONHSAG020075
l027686	chr16:81053497-81087651:-	NONHSAG020091
l027696	chr16:81738247-81962693:+	NONHSAG020107
l027719	chr16:84192557-84197053:-	NONHSAG020153
l027720	chr16:84255269-84261049:-	NONHSAG020155
l027769	chr16:86473637-86509099:-	NONHSAG020218

Unified node ID	Genomic coordinates	Matched evidence ID[1]
l027773	chr16:86564914-86567845:-	NONHSAG020223
l027798	chr16:87492554-87518834:+	NONHSAG020252
l027803	chr16:87696484-87765992:-	NONHSAG020259
l027805	chr16:87766087-87815326:-	NONHSAG020260
l027811	chr16:88076949-88101504:-	NONHSAG020274
l027812	chr16:88086852-88095447:+	NONHSAG020273
l027815	chr16:88177288-88187003:+	NONHSAG020277
l027816	chr16:88191485-88193667:-	NONHSAG020278
l027818	chr16:88234784-88302511:+	NONHSAG020281
l027830	chr16:88708955-88710437:-	NONHSAG020298
l027833	chr16:88731179-88741425:+	NONHSAG020304
l027835	chr16:88742766-88745748:+	NONHSAG020307
l027838	chr16:88856219-88861111:+	NONHSAG020312
l027846	chr16:89113174-89115279:-	NONHSAG020321
l027848	chr16:89166382-89169147:+	NONHSAG020325
l027858	chr16:89291154-89300959:+	AC137932.5;NONHSAG020339
l027873	chr16:89711855-89718165:+	NONHSAG020361
l027876	chr16:89816776-89827965:+	NONHSAG020367
l027878	chr16:89873569-89920973:+	NONHSAG020372
l027879	chr16:89898302-89923173:-	NONHSAG020374
l027884	chr16:89972585-89996623:+	NONHSAG020382
l027897	chr17:212388-386254:-	NONHSAG020403
l027943	chr17:1710719-1717174:-	NONHSAG020474
l027945	chr17:1725747-1738585:-	NONHSAG020476
l028032	chr17:4904211-4934693:-	NONHSAG020600

Unified node ID	Genomic coordinates	Matched evidence ID[1]
l028041	chr17:5019213-5030970:-	NONHSAG020616
l028075	chr17:6762062-6776116:-	PRAL
l028114	chr17:7674929-7687482:-	NONHSAG020729
l028115	chr17:7686070-7703502:+	NONHSAG020731
l028129	chr17:8172456-8224043:-	NONHSAG020759
l028130	chr17:8176811-8183457:+	NONHSAG020755
l028132	chr17:8224814-8248058:-	NONHSAG020760;NONHSAG020762
l028139	chr17:8314348-8321067:-	NONHSAG020771
l028171	chr17:10291296-10625540:+	NONHSAG072945
l028208	chr17:13756076-14069495:-	NONHSAG020869
l028248	chr17:16438766-16480249:+	NONHSAG020957;SNHG29
l028268	chr17:17011909-17015052:-	NONHSAG020990
l028282	chr17:17591427-17632141:+	NONHSAG074052
l028296	chr17:18172478-18184791:-	NONHSAG021042
l028314	chr17:18661936-18681846:-	NONHSAG021072;ZNF286B
l028389	chr17:20868432-21002276:+	NONHSAG073025
l028471	chr17:28573116-28574243:+	NONHSAG021307
l028472	chr17:28598789-28617377:+	NONHSAG021309
l028474	chr17:28633205-28635952:+	NONHSAG021315
l028488	chr17:28749730-28750079:-	NONHSAG021335
l028495	chr17:28897737-28899402:+	NONHSAG021342
l028498	chr17:28926274-28954928:+	NONHSAG021347
l028509	chr17:29573474-29594054:-	NONHSAG021370
l028524	chr17:30236961-30274429:+	NONHSAG021390
l028528	chr17:30536423-30565576:-	NONHSAG021400

Unified node ID	Genomic coordinates	Matched evidence ID[1]
l028531	chr17:30576408-31093258:+	NONHSAG021408
l028533	chr17:30726304-30727564:-	NONHSAG021410
l028543	chr17:31061719-31096045:-	NONHSAG021431
l028544	chr17:31094926-31382116:+	NONHSAG021432
l028545	chr17:31232069-31273617:-	NONHSAG021433
l028572	chr17:32324430-32360700:+	NONHSAG021471
l028623	chr17:35568049-35574900:+	NONHSAG021557;SNHG30
l028630	chr17:35809481-35864615:+	NONHSAG021567
l028631	chr17:35816716-35830293:-	NONHSAG021566
l028639	chr17:36115692-36177516:+	CTB-91J4.1;NONHSAG021584
l028674	chr17:37609572-37689790:+	NONHSAG021638
l028675	chr17:37741400-37748677:+	NONHSAG021641
l028718	chr17:39401781-39410725:+	NONHSAG021698
l028761	chr17:40801423-40809517:+	NONHSAG021760
l028772	chr17:41549605-41567364:-	NONHSAG021780
l028799	chr17:42313323-42388568:-	NONHSAG021820
l028818	chr17:42683186-42699466:-	NONHSAG021846
l028837	chr17:43125582-43153670:+	NONHSAG021874
l028841	chr17:43216940-43322791:-	NONHSAG021878
l028843	chr17:43325599-43389473:-	NONHSAG021882
l028852	chr17:43679340-43706709:+	LINC02594;NONHSAG021893
l028865	chr17:44115911-44120595:+	NONHSAG021909
l028911	chr17:45231048-45241957:-	NONHSAG021976
l028918	chr17:45396931-45397477:+	NONHSAG021981
l028931	chr17:45799389-45895630:-	NONHSAG022009

Unified node ID	Genomic coordinates	Matched evidence ID[1]
l028948	chr17:46982785-47100323:-	NONHSAG022030
l028966	chr17:47879471-47941411:-	NONHSAG022058
l028985	chr17:48543550-48606415:+	NONHSAG022078
l028988	chr17:48628674-48634933:+	HOXB-AS4;NONHSAG022088
l028991	chr17:48723168-48725458:+	NONHSAG022094
l028996	chr17:48873809-48908983:-	NONHSAG022103
l029002	chr17:48993890-49000223:-	NONHSAG022110
l029010	chr17:49361149-49476988:+	NONHSAG022125;NONHSAG022127
l029031	chr17:50156373-50181256:-	NONHSAG022162
l029122	chr17:57832640-57836126:+	NONHSAG022311
l029139	chr17:58324471-58415766:+	NONHSAG022327
l029140	chr17:58329667-58332524:-	NONHSAG022328
l029145	chr17:58514722-58583963:+	NONHSAG022335;SEPTIN4-AS1
l029147	chr17:58692572-58735611:+	NONHSAG022339
l029175	chr17:60122293-60141635:-	NONHSAG022378
l029188	chr17:61356838-61411555:-	NONHSAG073786
l029236	chr17:64145207-64153168:+	NONHSAG022495;SNHG25
l029245	chr17:64749522-64797300:-	NONHSAG022510
l029272	chr17:67032408-67033290:-	NONHSAG022551
l029289	chr17:67993718-67996434:-	NONHSAG022579
l029300	chr17:68413622-68524949:+	NONHSAG022595;NONHSAG022597
l029335	chr17:72221071-72641375:-	NONHSAG022655
l029362	chr17:74188213-74215551:-	NONHSAG022690
l029394	chr17:75271368-75275503:+	NONHSAG022743
l029399	chr17:75394062-75394963:+	NONHSAG022750

Unified node ID	Genomic coordinates	Matched evidence ID[1]
l029424	chr17:75979230-76005999:+	TEN1-CDK3
l029431	chr17:76138500-76154650:+	NONHSAG022806
l029441	chr17:76527585-76792366:+	NONHSAG073380;SNHG16
l029449	chr17:76779686-76782221:-	NONHSAG022836
l029456	chr17:77086715-77099442:+	NONHSAG022845
l029462	chr17:77256953-77282102:-	NONHSAG022851;SEPTIN9-DT
l029486	chr17:78315715-78351181:+	AC061992.2;NONHSAG073406
l029487	chr17:78357450-78376635:+	AC061992.2;NONHSAG022888
l029492	chr17:78484867-78503056:+	NONHSAG022893
l029510	chr17:79707265-79712317:+	NONHSAG073419
l029544	chr17:80960765-80961713:-	NONHSAG022965
l029548	chr17:81017782-81034881:-	BAIAP2-DT;NONHSAG022972
l029552	chr17:81135770-81136256:+	NONHSAG022980
l029554	chr17:81197392-81201081:+	NONHSAG022982
l029574	chr17:81514046-81528310:+	NONHSAG023003
l029602	chr17:81927828-81933591:+	NONHSAG023049
l029605	chr17:81937414-81956167:+	AC137723.5;NONHSAG023051
l029619	chr17:82212927-82217694:+	NONHSAG023073
l029680	chr18:812387-815669:+	NONHSAG023175
l029717	chr18:3466249-3487033:+	NONHSAG075363
l029719	chr18:3593737-3598352:+	NONHSAG023237
l029772	chr18:9102629-9285985:+	RP11-21J18.1
l029812	chr18:11810405-11811484:-	NONHSAG023375
l029822	chr18:12081539-12085267:+	NONHSAG023387
l029826	chr18:12228875-12234484:-	NONHSAG023392

Unified node ID	Genomic coordinates	Matched evidence ID[1]
l029890	chr18:21379926-21451062:-	NONHSAG023494
l029900	chr18:22087582-22171660:-	GATA6-AS1;NONHSAG023512
l029918	chr18:23957745-23982644:-	NONHSAG023553
l029936	chr18:25055599-25056760:+	NONHSAG023579
l029949	chr18:26655504-27190698:+	AQP4-AS1;NONHSAG074661
l029950	chr18:26668231-26741798:-	NONHSAG023598
l029955	chr18:27223441-27346184:+	LINC01908;NONHSAG023607
l029960	chr18:27921458-27966691:+	NONHSAG023614
l029974	chr18:31101354-31175412:+	NONHSAG023630;RP11-408H20.2
l029977	chr18:31327574-31426988:-	NONHSAG023633
l029978	chr18:31542145-31556911:-	NONHSAG023635
l030000	chr18:32833416-32938316:+	NONHSAG023661
l030006	chr18:34222964-34224761:+	NONHSAG023668
l030049	chr18:39840295-39924868:-	LINC01901;NONHSAG023740
l030089	chr18:47077360-47091280:-	RP11-49K24.4
l030119	chr18:49487327-49491878:+	NONHSAG023837
l030138	chr18:51028527-51085045:+	NONHSAG023864
l030148	chr18:53480834-53568283:-	NONHSAG023885
l030150	chr18:53577948-53581183:-	LINC01917
l030174	chr18:57054558-57072119:-	NONHSAG023930
l030198	chr18:58672612-58780225:-	NONHSAG023964
l030291	chr18:70335928-70337310:-	NONHSAG024117
l030311	chr18:72863303-72881404:+	NONHSAG024141
l030313	chr18:73324756-73392162:+	LINC02582;NONHSAG024146
l030330	chr18:74583479-74598583:-	NONHSAG024171

Unified node ID	Genomic coordinates	Matched evidence ID[1]
l030372	chr18:77971293-77993727:-	NONHSAG024224
l030387	chr18:79576031-79601711:+	NONHSAG024244
l030415	chr19:305570-325706:+	NONHSAG024282
l030452	chr19:1038726-1039064:-	NONHSAG024332
l030458	chr19:1177557-1228431:+	NONHSAG024340;NONHSAG024341
l030470	chr19:1321224-1322864:+	NONHSAG024354
l030474	chr19:1383526-1395584:+	NONHSAG024361
l030475	chr19:1385158-1389665:-	NONHSAG024362
l030476	chr19:1392169-1396467:-	NONHSAG024364
l030489	chr19:1573595-1592865:-	NONHSAG024381
l030496	chr19:1815248-1815873:+	NONHSAG024391
l030497	chr19:1822088-1824545:+	NONHSAG024393
l030499	chr19:1852381-1853622:+	NONHSAG024395
l030522	chr19:2287163-2287943:-	NONHSAG024428
l030605	chr19:4769104-4774021:+	NONHSAG024540
l030606	chr19:4769567-4791554:-	NONHSAG024542
l030669	chr19:7388910-7395049:-	NONHSAG024637
l030693	chr19:7918632-7920295:-	NONHSAG024668
l030696	chr19:7925404-7933929:+	NONHSAG024671
l030701	chr19:7959122-7960012:+	NONHSAG024676
l030703	chr19:8065401-8472191:-	NONHSAG024681
l030734	chr19:9538715-9540740:+	NONHSAG024741
l030766	chr19:10385805-10933533:+	NONHSAG024809
l030772	chr19:11010916-11016011:-	NONHSAG024810
l030775	chr19:11197617-11221809:+	NONHSAG024818

Unified node ID	Genomic coordinates	Matched evidence ID[1]
l030777	chr19:11295714-11324441:-	NONHSAG024826
l030841	chr19:12796822-12801849:+	NONHSAG024903
l030842	chr19:12825710-12833097:+	NONHSAG024905
l030844	chr19:12880861-12889980:+	NONHSAG024908
l030849	chr19:12944117-12944487:-	NONHSAG024913
l030856	chr19:13116821-13118135:+	NONHSAG024921
l030858	chr19:13139616-13141147:-	NONHSAG024923
l030861	chr19:13153070-13154193:-	NONHSAG024925
l030894	chr19:14939433-16643942:+	NONHSAG024979;NONHSAG025011
l030980	chr19:18522515-18532726:+	NONHSAG025135
l030983	chr19:18557774-18571689:-	NONHSAG025137
l030995	chr19:18940198-18947492:+	HOMER3-AS1;NONHSAG025157
l031015	chr19:19516241-19528199:+	NONHSAG025185
l031094	chr19:22665551-22717311:-	NONHSAG025319
l031107	chr19:23251069-23416101:-	NONHSAG025352
l031200	chr19:33299933-33302524:+	NONHSAG025483
l031201	chr19:33301753-33313294:+	CEBPA-DT;NONHSAG025485
l031284	chr19:35769143-35771028:-	NONHSAG025609
l031358	chr19:37855338-37884418:+	NONHSAG025712
l031375	chr19:38526953-38528555:-	NONHSAG025743
l031376	chr19:38535515-38537245:-	NONHSAG025744
l031456	chr19:41221425-41222051:-	CTD-2195B23.3
l031461	chr19:41361084-41364309:-	NONHSAG025864
l031463	chr19:41397807-41425002:+	NONHSAG025865
l031466	chr19:41454168-41500644:-	NONHSAG025867

Unified node ID	Genomic coordinates	Matched evidence ID[1]
l031492	chr19:42424383-42425071:-	NONHSAG025907
l031508	chr19:43360684-43369075:-	NONHSAG025934
l031512	chr19:43574637-43575618:+	NONHSAG025939
l031530	chr19:44023117-44025262:-	NONHSAG025964
l031556	chr19:44737604-44747649:+	NONHSAG025997
l031595	chr19:45764784-45779526:+	DM1-AS;NONHSAG026053
l031617	chr19:46605816-46610779:+	NONHSAG026089
l031618	chr19:46608984-46610873:-	NONHSAG026091
l031625	chr19:46748025-46777430:+	NONHSAG026103
l031660	chr19:48145907-48147574:+	NONHSAG026153
l031679	chr19:48619271-48630120:-	NONHSAG026176
l031697	chr19:48955666-48961011:+	NONHSAG026205
l031702	chr19:49017495-49020523:-	CTB-60B18.10
l031746	chr19:49852525-49859569:-	NONHSAG026274
l031747	chr19:49859881-49878459:-	AC018766.4
l031795	chr19:51387084-51420665:+	NONHSAG026364
l031808	chr19:51853422-51900531:+	NONHSAG026386
l031933	chr19:55312028-55312495:-	NONHSAG026575
l031951	chr19:55661900-55674715:-	NONHSAG026599
l031972	chr19:56477249-56500666:+	NONHSAG026631
l031993	chr19:57304304-57308562:+	NONHSAG026661
l032040	chr19:58346853-58362751:-	NONHSAG026720
l032041	chr19:58347202-58361730:+	A1BG-AS1;NONHSAG026721
l032052	chr19:58499490-58512413:+	NONHSAG026737
l032056	chr19:58558590-58605223:+	MZF1-AS1;NONHSAG026743

Unified node ID	Genomic coordinates	Matched evidence ID[1]
l032092	chr20:2206494-2258057:+	NONHSAG031123
l032185	chr20:10637683-10673999:-	NONHSAG031261
l032271	chr20:19705639-20002457:+	NONHSAG031385
l032297	chr20:21511446-21513711:+	NKX2-2-AS1;NONHSAG031425
l032311	chr20:22547670-22578877:-	NONHSAG031452
l032312	chr20:22587236-22607971:-	LNCNEF;NONHSAG031453
l032314	chr20:22668479-22685394:-	LINC01747;NONHSAG031456
l032330	chr20:23354803-23358214:-	NONHSAG031481
l032384	chr20:30273264-30290066:-	LINC01597;NONHSAG031563;RP4-610C12.4
l032386	chr20:30289716-30417848:+	NONHSAG031565
l032397	chr20:31567706-31573263:-	NONHSAG031581
l032399	chr20:31664460-31723926:-	NONHSAG031584
l032441	chr20:33786424-33812320:-	NONHSAG031636
l032483	chr20:36042279-36051043:-	NONHSAG031696;NORAD
l032597	chr20:44966493-45084214:+	NONHSAG031853
l032613	chr20:45892693-45893419:-	NONHSAG081993
l032630	chr20:46709648-46736347:+	NONHSAG031900
l032637	chr20:47352416-47354634:+	NONHSAG031913
l032666	chr20:49276738-49441654:+	NONHSAG081583;ZFAS1
l032672	chr20:50040706-50041629:-	NONHSAG031991
l032720	chr20:53905874-53962971:+	NONHSAG032063
l032758	chr20:57710155-57718287:+	NKILA;NONHSAG032126
l032840	chr20:62657140-62666725:-	NONHSAG032247
l032856	chr20:63101291-63104387:+	NONHSAG032267
l032879	chr20:63626147-63629971:+	MHENCR;NONHSAG032297

Unified node ID	Genomic coordinates	Matched evidence ID[1]
l032883	chr20:63695339-63696412:-	NONHSAG032302
l032889	chr20:63744595-63747294:+	NONHSAG032309
l032891	chr20:63859936-63865874:-	NONHSAG032312
l032967	chr21:15928295-16645495:+	NONHSAG083289
l033027	chr21:24304549-24353571:-	NONHSAG032537
l033039	chr21:25560398-25582739:+	NONHSAG032560
l033105	chr21:32277862-32284702:+	MIS18A-AS1;NONHSAG032664
l033117	chr21:32728105-32747072:+	NONHSAG032682
l033142	chr21:33903453-33915854:-	NONHSAG032724
l033147	chr21:34353098-34375809:-	NONHSAG032735
l033150	chr21:34446687-34512214:-	NONHSAG032737
l033155	chr21:34745756-34785180:+	NONHSAG082891
l033156	chr21:34787800-36004667:-	NONHSAG032748;NONHSAG032750
l033323	chr21:44200254-44207400:-	AP001058.3;NONHSAG083209
l033327	chr21:44339121-44342412:+	NONHSAG110858
l033328	chr21:44401910-44425596:-	NONHSAG033027;TRPM2-AS
l033352	chr21:44999207-45004728:-	NONHSAG033061
l033377	chr21:45972812-45974956:+	NONHSAG033100
l033380	chr21:46033527-46039824:+	NONHSAG033105
l033381	chr21:46040984-46057643:+	AP001476.2;NONHSAG033107
l033392	chr21:46228393-46284359:+	MCM3AP-AS1;NONHSAG033121
l033421	chr22:16601351-16698742:+	NONHSAG033168;TPTEP1
l033423	chr22:16654065-16679493:-	ANKRD62P1-PARP4P3;NONHSAG033170
l033424	chr22:16676015-16694648:-	KB-7G2.8
l033451	chr22:17787648-18024561:-	NONHSAG033216

Unified node ID	Genomic coordinates	Matched evidence ID[1]
l033455	chr22:18029384-18043077:+	NONHSAG033224
l033481	chr22:18969859-19031605:+	NONHSAG033254
l033489	chr22:19110287-19136530:-	NONHSAG083883
l033490	chr22:19121463-19135657:+	NONHSAG033263
l033493	chr22:19171394-19185045:+	LINC01311;NONHSAG033266
l033516	chr22:20058029-20070569:-	NONHSAG033298
l033522	chr22:20198728-20212221:-	XXbac-B444P24.8
l033530	chr22:20522069-20523871:-	NONHSAG033328
l033535	chr22:20700812-20706838:+	NONHSAG033334
l033538	chr22:20889205-20891214:-	NONHSAG083894
l033540	chr22:20957088-20965072:+	NONHSAG033342
l033546	chr22:21001874-21010374:+	NONHSAG033348
l033550	chr22:21031353-21044117:+	NONHSAG033352
l033562	chr22:21467915-21469172:+	NONHSAG033368
l033578	chr22:21938236-21977632:+	NONHSAG033386
l033581	chr22:22026032-22923031:+	LL22NC03-23C6.13;NONHSAG033390
l033582	chr22:22031159-22922981:-	NONHSAG033435
l033586	chr22:23182044-23253833:+	NONHSAG033459
l033594	chr22:23433527-23435072:+	NONHSAG033471
l033598	chr22:23536880-23547797:+	NONHSAG083615
l033601	chr22:23573122-23717423:-	NONHSAG033480
l033627	chr22:24598053-24599398:+	NONHSAG033528
l033630	chr22:24645165-24659147:-	AP000356.2
l033672	chr22:26646427-26853001:+	NONHSAG033589
l033693	chr22:27919367-28008581:+	NONHSAG033611

Unified node ID	Genomic coordinates	Matched evidence ID[1]
l033716	chr22:29603574-29668018:+	NONHSAG033645
l033742	chr22:30969157-30985225:+	NONHSAG033691
l033756	chr22:31399603-31528740:+	NONHSAG033716
l033768	chr22:31970717-32037995:+	LINC02558;NONHSAG033735
l033775	chr22:32327063-32343106:-	NONHSAG033748
l033812	chr22:35744077-35809899:-	NONHSAG033810
l033851	chr22:37291177-37335523:-	NONHSAG033862
l033870	chr22:37947831-38002889:+	NONHSAG033896
l033872	chr22:38032164-38034229:-	NONHSAG033898
l033874	chr22:38057179-38073941:-	NONHSAG033899
l033895	chr22:38705741-38738990:+	NONHSAG033937
l033898	chr22:38739002-38749042:+	NONHSAG033940
l033939	chr22:41169189-41197499:-	NONHSAG034006
l033947	chr22:41551372-41560909:-	NONHSAG034014
l033951	chr22:41666540-41667975:-	NONHSAG034021
l033958	chr22:41922022-41923100:-	CTA-250D10.23;NONHSAG034032
l034095	chr22:49718052-49724506:-	NONHSAG034217
l034124	chr22:50523925-50524780:+	NONHSAG083859
l034145	chrX:1392280-1416343:+	ASMTL-AS1;NONHSAG103259
l034210	chrX:12902816-12943300:-	NONHSAG054024
l034216	chrX:13266031-13308637:-	LINC02154;NONHSAG054038
l034325	chrX:31097676-31169557:+	NONHSAG054212
l034326	chrX:31097676-33339609:-	NONHSAG054213;NONHSAG054215;NONHSAG054216
l034327	chrX:31261107-31323630:+	NONHSAG054214
l034377	chrX:41345581-41345876:-	NONHSAG054301

Unified node ID	Genomic coordinates	Matched evidence ID[1]
l034400	chrX:45745209-45852527:-	NONHSAG104186
l034411	chrX:46545489-46559681:+	NONHSAG054360
l034456	chrX:48636632-48801376:-	NONHSAG054432
l034490	chrX:50365408-50470825:-	DGKK
l034621	chrX:70452955-70455994:-	NONHSAG054699
l034629	chrX:71122432-71198507:-	NONHSAG054716;NONHSAG054718
l034630	chrX:71137187-71137392:+	NONHSAG054717
l034653	chrX:72777607-72779097:+	FAM226B;NONHSAG103457
l034661	chrX:73817774-73852754:-	NONHSAG054780
l034663	chrX:73943933-74070832:+	JPX;NONHSAG054791
l034664	chrX:73946554-74293574:-	NONHSAG103817
l034677	chrX:74408535-74420767:-	NONHSAG054807
l034696	chrX:77599731-77633335:-	NONHSAG054832
l034789	chrX:102599511-102917367:+	NONHSAG055008
l034837	chrX:108736008-108739831:+	NONHSAG055088
l034842	chrX:109628868-109629579:+	NONHSAG055092
l034873	chrX:115840797-115908066:+	DANT1;NONHSAG055151
l034931	chrX:123923110-124031048:-	NONHSAG055258
l034985	chrX:134540008-134549645:-	NONHSAG055350
l035025	chrX:138711451-138716618:+	NONHSAG055418
l035044	chrX:141502848-141649939:+	NONHSAG055450
l035105	chrX:153494952-153509546:+	NONHSAG055546
l035113	chrX:153735625-153766488:-	NONHSAG055558
l035118	chrX:153861513-153886173:-	NONHSAG055565
l035125	chrX:154032373-154137103:-	NONHSAG055577

Unified node ID	Genomic coordinates	Matched evidence ID[1]
l035127	chrX:154348534-154350196:-	NONHSAG055582

Note: [1]The specific gene identifiers or symbols (belonging to the unified node) that were successfully matched against the dbEssLnc2.0 database.

Table S7 - Mouse essential lncRNA genes in the benchmarking dataset

Unified node ID	Genomic coordinates	Matched evidence ID[1]
l000368	chr1:42707071-42713708:-	NONMMUG000508
l001532	chr1:161034421-161038539:+	Gas5;NONMMUG002215
l001547	chr1:162217622-162225550:+	Dnm3os;NONMMUG002239
l003011	chr2:74748421-74762897:-	Haglr;NONMMUG023454
l003961	chr2:147944739-148040203:-	9030622O22Rik
l004853	chr3:34762518-34782392:+	Gm38509;NONMMUG026355
l007173	chr4:89295044-89518621:+	Gm12610;NONMMUG029813
l007422	chr4:109402279-109407100:-	NONMMUG030202;Ttc39aos1
l009617	chr5:112213127-112229395:-	Miat;NONMMUG033401
l010369	chr6:6820188-6871593:-	Dlx6os1
l010565	chr6:31063747-31220488:-	Lncpint;NONMMUG034886
l013112	chr7:73539202-73561696:-	NONMMUG038355
l013919	chr7:139957004-139958039:-	Gm2044
l013980	chr7:143203457-143298451:-	Kcnq1ot1;NONMMUG039685
l014935	chr8:85994243-85996662:+	Hm629797;NONMMUG041158
l015304	chr8:121054792-121083111:-	Fendrr;NONMMUG041781
l020930	chr12:109540995-109571729:+	NONMMUG009962
l022718	chr14:64587286-64593961:+	NONMMUG013211
l025226	chr17:12741310-12860182:+	Airn;NONMMUG017485
l025462	chr17:29057473-29078962:-	NONMMUG017913
l027077	chr19:5824551-5845878:-	NONMMUG020673;Neat1
l028501	chrX:103431516-103484958:+	NONMMUG045568;Tsix
l028502	chrX:103460365-103483254:-	NONMMUG045569
l028503	chrX:103493555-103565696:+	Jpx;NONMMUG045574

Note: [1]The specific gene identifiers or symbols (belonging to the unified node) that were successfully matched against the dbEssLnc2.0 database.

Table S8 - Genomic and epigenomic features in ELE

Feature name	Description	Source	Molecule
GC_content(%)	The content of G and C in lncRNA gene sequence	NONCODE and Ensembl	LncRNA genes
CpG_islands	The number of consecutively repeated CG clusters (CGCG, CGCGCG…) in lncRNA gene sequence	NONCODE and Ensembl	LncRNA genes
CpG_count	The number of continuous CG dinucleotides in lncRNA gene sequence	NONCODE and Ensembl	LncRNA genes
Length	The length of lncRNA gene sequence	NONCODE and Ensembl	LncRNA genes
phyloP_mean_score	The mean value of phyloP score within the range of lncRNA genes (phyloP100way for human and phyloP60way for mouse)	UCSC	LncRNA genes
phyloP_max_score	The median value of phyloP score within the range of lncRNA genes (phyloP100way for human and phyloP60way for mouse)	UCSC	LncRNA genes
phastCons_mean_score	The mean value of phastCons score within the range of lncRNA genes (phastCons100way for human and phastCons60way for mouse)	UCSC	LncRNA genes
phastCons_max_score	The median value of phastCons score within the range of lncRNA genes (phastCons100way for	UCSC	LncRNA genes

Feature name	Description	Source	Molecule
	human and phastCons60way for mouse)		
DHS_peak_counts	The number of DNase hypersensitive peaks on lncRNA geness	ENCODE	LncRNA genes
DHS_max_signalValue	The maximum signal value of DNase supersensitive peak on lncRNA genes	ENCODE	LncRNA genes
H3K4me3_peak_counts	The number of H3K4me3 peaks on lncRNA genes	ENCODE	LncRNA genes
H3K4me3_max_signalValue	The maximum signal value of H3K4me3 peak on lncRNA genes	ENCODE	LncRNA genes
H3K9me3_peak_counts	The number of H3K9me3 peaks on lncRNA genes	ENCODE	LncRNA genes
H3K9me3_max_signalValue	The maximum signal value of H3K9me3 peak on lncRNA genes	ENCODE	LncRNA genes
H3K27ac_peak_counts	The number of H3K27ac peaks on lncRNA genes	ENCODE	LncRNA genes
H3K27ac_max_signalValue	The maximum signal value of H3K27ac peak on lncRNA genes	ENCODE	LncRNA genes
H3K36me3_peak_counts	The number of H3K36me3 peaks on lncRNA genes	ENCODE	LncRNA genes
H3K36me3_max_signalValue	The maximum signal value of H3K36me3 peak on lncRNA gene	ENCODE	LncRNA genes
H3K27me3_peak_counts	The number of H3K27me3 peaks on lncRNA genes	ENCODE	LncRNA genes
H3K27me3_max_signalValue	The maximum signal value of H3K27me3 peak on lncRNA genes	ENCODE	LncRNA genes
pme_exp	The pme_TPM of each gene.	ENCODE	Protein-coding genes

Feature name	Description	Source	Molecule
GO_Term_Count	The number of Go Term of each gene.	Ensembl BioMart for human and MGI for mouse	Protein-coding genes
ortholog_count	The number of orthologous genes of protein-coding genes.	Ensembl REST API for both human and mouse.	Protein-coding genes

Table S9 - ENCODE accession numbers in this work

Tissue	Feature	Accesses
Human left ventricle	DHS	ENCFF756YUX
Human left ventricle	H3K4me3	ENCFF629KFL
Human left ventricle	H3K27ac	ENCFF602BBS
Human left ventricle	H3K27mes	ENCFF291FSU
Human left ventricle	H3K36me3	ENCFF076IWC
Human left ventricle	H3K9me3	ENCFF910VAC
Human left ventricle	Expression	ENCFF789UOH
Human left lung	DHS	ENCFF527WRB
Human left lung	H3K4me3	ENCFF444RGS
Human left lung	H3K27ac	ENCFF339VLN
Human left lung	H3K27mes	ENCFF617AEQ
Human left lung	H3K36me3	ENCFF590KXQ
Human left lung	H3K9me3	ENCFF327QLD
Human left lung	Expression	ENCFF489CHJ
Human stomach	DHS	ENCFF248XHU
Human stomach	H3K4me3	ENCFF039SDG
Human stomach	H3K27ac	ENCFF541IKF
Human stomach	H3K27mes	ENCFF561MQR
Human stomach	H3K36me3	ENCFF948KTL
Human stomach	H3K9me3	ENCFF563GAC
Human stomach	Expression	ENCFF952ZOE
Mouse heart	DHS	ENCFF017NJZ
Mouse heart	H3K4me3	ENCFF992IYB
Mouse heart	H3K27ac	ENCFF325TQE
Mouse heart	H3K27mes	ENCFF904XXX
Mouse heart	H3K36me3	ENCFF614WZG
Mouse heart	H3K9me3	ENCFF411LJG
Mouse heart	Expression	ENCFF868TGE
Mouse lung	DHS	ENCFF561HDY
Mouse lung	H3K4me3	ENCFF606VSQ
Mouse lung	H3K27ac	ENCFF187NTP
Mouse lung	H3K27mes	ENCFF912VDC
Mouse lung	H3K36me3	ENCFF936YCL
Mouse lung	H3K9me3	ENCFF651FZP
Mouse lung	Expression	ENCFF633HRL
Mouse forebrain	DHS	ENCFF544FGH
Mouse forebrain	H3K4me3	ENCFF307IKE
Mouse forebrain	H3K27ac	ENCFF643PLF
Mouse forebrain	H3K27mes	ENCFF085OWP
Mouse forebrain	H3K36me3	ENCFF857VRR
Mouse forebrain	H3K9me3	ENCFF721TNV
Mouse forebrain	Expression	ENCFF145PTV
